# Quantum Health Accelerator^®^ Ameliorates CFA-Induced Animal Model of Rheumatoid Arthritis: Investigating the Role of Immunomodulatory and Anti-Oxidative Effects

**DOI:** 10.3390/brainsci15030232

**Published:** 2025-02-23

**Authors:** Ali Akbar Mohaddes, Mohammad Ali Saatchi, Marziyeh Afshari Chamanabadi, Saeed Saatchi, Sadra Rostami, Vahid Reza Askari

**Affiliations:** 1 International Group of Ali Akbar Mohaddes Company, License NO 1090645, Dubai 35360-97797, United Arab Emirates; aliakbarmohaddes@intgraam.com (A.A.M.); mohammadalisaatchi@intgraam.com (M.A.S.); marziyeafshari@intgraam.com (M.A.C.); saeedsaatchi@intgraam.com (S.S.); sadrarostami@intgraam.com (S.R.); 2 Pharmacological Research Center of Medicinal Plants, Mashhad University of Medical Sciences, Mashhad 9177948564, Iran

**Keywords:** water enriched with vital bio-quantum information, rheumatoid arthritis, neuropathic pain, inflammation, oxidative stress, immune-modulation

## Abstract

**Introduction:** Rheumatoid arthritis (RA) is a systemic inflammatory and autoimmune disease characterized by joint swelling, pain, damage to the cartilage, and disability. In the present study, we aimed to evaluate the anti-oxidant, anti-inflammatory, and immune-modulatory properties of Quantum Health Accelerator^®^ as water enriched with vital bio-quantum information/energy (EW) following complete Freund’s adjuvant (CFA)-induced RA in rats. **Methods:** Forty adult male Wistar rats (180–220 g) were divided into five groups. Arthritis was induced on day one using a single subcutaneous injection of CFA into the left hind footpad of the rat. Rats were assigned to receive methotrexate (MTX, 2 mg/kg/week, intraperitoneally), EW (orally, instead of normal water ad libitum), or their combination for 29 days. The anti-RA activities were determined by paw edema, joint diameter, arthritis score, and several nociceptive behavioral tests (thermal hyperalgesia, cold allodynia, and tactile allodynia). The levels of inflammatory (TNF-α, CRP, RF, and anti-CCP), anti-inflammatory (IL-10), and oxidative stress (NO, MDA, and GSH) markers were measured in serum. In addition, the levels of IFN-γ, IL-4, IL-17, and TGF-β were assessed in the spleen-isolated lymphocytes. **Results:** We found that treatment with MTX, EW, and their combination remarkably ameliorated thermal hyperalgesia, cold allodynia, and tactile allodynia results following CFA-induced RA in rats. In addition, EW also notably attenuated arthritis score, joint diameter, inflammatory cytokines, and oxidative markers while propagating anti-inflammatory and anti-oxidative mediators. **Conclusions**: We reveal that EW possesses anti-arthritic effects, possibly through anti-oxidative, anti-inflammatory, and immunomodulatory properties. Collectively, EW may be a promising therapeutic agent for treating RA.

## 1. Introduction

Rheumatoid arthritis (RA) is a systemic inflammatory and autoimmune disease characterized by joint swelling, pain, damage to the cartilage, and disability [1]. When left untreated, the prolonged chronic inflammation may lead to disease progression, causing severe irreversible disabilities, disrupting quality of life, and enhancing mortality rate [2,3]. It has been noticed that the imbalance between pro-inflammatory and anti-inflammatory mediators triggers auto-immunity and chronic inflammation in RA [4,5]. In fact, the immune system attacks healthy tissues and cells other than invasive pathogens and infections due to chronic inflammation, resulting in joint injuries in RA patients [6,7].

The therapeutic regimen of RA patients primarily consists of nonsteroidal anti-inflammatory drugs (NSAIDs), glucocorticoids, methotrexate, and newer remedies such as tumor necrosis factor-α (TNF-α) inhibitors and Janus kinase inhibitors [8]. NSAIDS such as naproxen, indomethacin, and celecoxib are widely used in RA patients in order to ameliorate pain and inflammation. However, they cannot prevent disease progression and have common side effects such as gastrointestinal bleeding and cardiovascular complications [9]. Furthermore, glucocorticoids are more robust anti-inflammatory drugs than NSAIDs. However, they have more significant adverse effects, especially when used in high doses and for extended periods. Therefore, glucocorticoids are mostly indicated in exacerbations of RA and for short durations [10].

Methotrexate (MTX) is an immunosuppressive agent that belongs to disease-modifying anti-rheumatic drugs (DMARDs). It is considered the most prevalent therapeutic of RA patients that propagates RA’s remission state. MTX inhibits the progression of joint damage and disabilities [2,3]. Accordingly, investigating possible new, safer, and more effective remedies for RA patients is required.

In recent decades, complementary and alternative medicine has attracted much attention in treating diseases. In this regard, some studies have been carried out on different types of water and their therapeutic effects. It has been emphasized that deuterium-depleted water possesses anti-aging, anti-oxidant, anti-inflammatory, antidepressant, anti-cancer, and hypoglycemic properties [11]. Moreover, natural thermal mineral water remarkably ameliorated functional capacity and pain in patients with knee osteoarthritis [12]. However, the anti-oxidant, anti-inflammatory, immunomodulatory, and anti-RA properties of water enriched with vital bio-quantum information (EW), Quantum Health Accelerator^®^, have not been investigated.

Regarding the idea of complementary therapies that define all living beings as a combination of energies that interact with other energy fields, techniques such as Fibonacci Carbon Unit for Bio-Quantum Data Absorption therapy have emerged in all parts of the world as an intervention in treatment and diagnostic methods. It is worth mentioning that the complementary approaches recognize the energy field (and not microbes or genes) as the factor ultimately determining a human’s state of health or illness. Consequently, the scientific study of this theory with specific principles through conventional methods seems inevitable.

In bio-quantum, quantum information refers to the quantum structures and states within biological cells and molecules that continuously exchange information with their surrounding environment [13,14,15]. This process includes vibrations, quantum waves, and even quantum correlations, essential for maintaining homeostasis and sustaining life. This concept aligns with the theory that classical mechanics cannot fully explain life, as quantum processes such as superposition, entanglement, and quantum tunneling are involved at the molecular level and even in more complex systems such as the brain and immune system. This suggests an informational connection between all living system components, interacting through a quantum information network [13,14,15,16].

Thus, from a bio-quantum perspective, “life” or the life force is not only a physical concept but also a complex informational process that flows through all levels of existence, from living cells to the human body and mind [17,18,19]. This approach could potentially expand our understanding of health, healing, and even the processes of aging and death. The idea that life, as a complex and open system, receives quantum information from the environment and simultaneously responds to it is consistent with the concept of quantum coherence in biological systems [20,21]. These responses are aligned with the “flow of time” in such a way that life can continuously adapt to new environmental conditions. Therefore, time in the bio-quantum is not just an external dimension but an essential internal element of the life process, existing and persisting in harmony with it [17,21,22].

From the bio-quantum perspective, life and the life force can be understood as a collection of complex processes, including the transfer of quantum information, vibrations, and energy frequencies. This theory enables us to look at how lifeless objects transform into living or “ensouled” entities, with vital information being transferred to them in the form of specific waves and frequencies [16,18,22,23,24]. The transfer of energy occurs through quantum waves and vibrations, which are transferred to objects at specific frequencies that can activate life processes in a living organism. These frequencies are specifically transmitted through water [16,24,25,26,27].

The frequencies that transfer quantum information/energy are generally in the low-frequency range, varying from 10^9^ Hz to 10^12^ Hz. Due to their longer wavelengths and lower energy, these waves allow biological systems to interact with them naturally [28,29,30,31,32]. This has been demonstrated through research on how quantum resonance occurs when the frequencies of quantum vibrations are in perfect harmony with the natural frequencies of water molecules, allowing water molecules to efficiently absorb and store information or energies [19,26,27,29,30,33,34,35,36,37]. These data or energies are transferred quantum mechanically and can activate essential functions such as cellular repair, immune system enhancement, and strengthening of the nervous system [28,29,31].

The wavelength and frequency of the information or energy transferred in the form of quantum waves are transmitted at specific wavelengths and frequencies. These waves are typically at the lower end of the electromagnetic frequency range, approximately between one billion and one trillion Hz. Due to their longer wavelengths and lower energy, these frequencies facilitate direct interaction with water molecules [28,29,31,38,39]. Due to hydrogen bonding, molecular vibrations in water can resonate with specific quantum waves. Specifically, asymmetric vibrations mean that certain quantum wave frequencies can affect the molecular structure of water and encode quantum information within it [40,41,42,43]. This phenomenon may lead to using water as a carrier of information or energy, a concept explored through quantum mechanical studies of water molecules [39,44,45,46,47]. The famous Schrödinger equation ($\hat{\Psi }$ H Ψ = E Ψ, E = energy, H = an observable, the Hamiltonian operator, Ψ = state vector of the quantum system) can show how the vibrational states of water molecules relate to quantum information [32,43,44,45,48].

Additionally, recent research into the quantum coherence of water in biological systems has revealed further insight into how quantum effects can influence molecular dynamics within living organisms [28,29,31,48].

## 2. Materials and Methods

The method of transferring bio-quantum information to water was achieved through the technology designed and developed by Fibonacci carbon units, which have the ability to absorb and retain the vital bio-quantum frequencies mentioned above from nature. These frequencies can then be transferred to enriched water. This technology is a confidential part of this study.

### 2.1. Created Technology (Quantum Carbon Embryo Named Fibonacci Atlantis)

The design and construction of the Fibonacci Atlantis quantum carbon unit were completed after 16,200 stages of technical failures and 122 million simulated scenarios using a genetic algorithm in COMSOL Multiphysics software version 6.2.339 (Figure S1). This sophisticated approach enabled the creation of a quantum carbon unit with optimized characteristics designed for enhanced coherence and stability, ensuring its functionality in quantum information transfer and related applications. In the simulated and hardware-produced unit, quantum biological information was received from an unknown source of life in nature at a wavelength of 300–3000 nm and an energy of 0.414–4.14 eV. This information was amplified 6400 times and transferred to the quantum tensile vibration frequency of water with a wavelength of 120 nm and energy of 14.4 eV. The information/energy was then added to the hexagonal water molecules.

A genetic algorithm was used to create a quantum carbon unit with optimized characteristics that were designed for enhanced coherence and stability, ensuring its functionality in quantum information transfer and related applications. The genetic algorithm is the most well-known optimization method used to determine the required layers in a composite structure with specific properties (Figure S1).

Water samples were prepared after being exposed to the quantum unit for 72 h and used in the current study.

### 2.2. Energy Measurement Method

This study used the ElectroPhotonic Imaging (EPI) technique to measure energy. This technique has been used in Eastern countries for centuries and is based on the Kirlian effect. This effect is foundational in an initial study of spectrum analysis as a special energy radiation phenomenon with the highest brightness, used for medical applications and disease interpretation. This technique separates electromagnetic fields into electric and magnetic spectra for observing gas discharge (GDV) using sensitive CCD detectors. It is employed to collect electro-photonic signals radiated from living organisms using high-frequency electrical stimulation. In the EPI imaging technique, after electrically stimulating the environment by sending high-frequency electromagnetic waves (mainly EHF, ranging from 40–70 GHz) and low intensities (typically 10 mW/cm^2^ or less), the dielectric constant of the environment was reduced, which decreased the resistance of the environment and triggered electrical discharge. This resulted in the observation of the GDV phenomenon (Gas Discharge Visualization). By collecting the electro-photons generated by the Kirlian phenomenon and analyzing their digital images, the electric field on the surface of an object or in the surrounding environment could be calculated through the radiation spectrum in GI (Glow Image) images. The energy stored in these images was then used to determine an individual’s or living organism’s physiological status.

The measurements were conducted using the Bio-Well Sputnik air sensor and a water probe. Energy variations in radiation during specific moments (such as dawn and twilight) were also measured according to the timing of radiation and the duration it was trapped in the structure. Then, when placed in various locations within the Fibonacci Carbon Unit for Bio-Quantum Data Absorption waveguide structure, the energy stored in different water samples (distilled, deionized, drinking, and physiological water) was measured using a Bio-Well water probe. Further results were used to determine the biological effects of the most active water within the Fibonacci Carbon Unit for Bio-Quantum Data Absorption structure (Figure S2).

Furthermore, chemical and microbial water test reports are shown in Figure S3.

In the next phase, this research assessed the potential anti-arthritis effects of the EW-Quantum Health Accelerator^®^ on Complete Freund’s Adjuvant (CFA) arthritis in rats.

### 2.3. Animals and Husbandry

Adult male rats (8–10 weeks old, weighing 180–220 g) were purchased from the Pasteur Institute of Iran. Then, they were randomly kept in the animal laboratory of the Department of Pharmaceutical Sciences in Persian Medicine, School of Persian and Complementary Medicine, Mashhad University of Medical Sciences, Mashhad, Iran. The rats were maintained in a hygienic environment at a controlled room temperature (22–25 °C) with a light/dark cycle (12 h on:12 h off) and 50% relative humidity. All animals had free access to clean, safe, filtered water and commercial standard rodent chow. All experiments and procedures were organized according to the *NIH’s Guideline for the Care and Use of Laboratory Animals*, accompanying approval from the *Animal Ethics Committee of Mashhad University of Medical Sciences* (approval no. 4000351, IR.MUMS.MEDICAL.REC.1400.297, date: 6 July 2021, Figure S4).

### 2.4. Arthritis Induction and Experimental Design

Arthritis was induced on day one using a 100 µL subcutaneous injection of complete Freund’s adjuvant (CFA, 0.1 mL containing 10 mg/mL mycobacterium) into the left hind footpad of the rat [49]. After that, rats were randomly divided into five experimental groups of eight. *Group one*. As assigned to the sham group, rats received 100 µL normal saline in lieu of CFA in the left hind paw and received the EW ad libitum. *Group two*. Assigned as the model group (RA), rats received 100 µL of CFA in the left hind paw and received the normal water ad libitum. *Group three*. Assigned as a positive control group with methotrexate (MTX), rats received 100 µL of CFA in the left hind paw and received normal water ad libitum and methotrexate (MTX, 2 mg/kg/week, intraperitoneally, [49]). *Group four*. Assigned to the EW group, rats received 100 µL of CFA in the left hind paw and received the EW instead of normal water ad libitum. *Group five*. Assigned to the EW and MTX combination group, rats received 100 µL of CFA in the left hind paw and received the EW instead of normal water ad libitum and MTX (2 mg/kg/week, intraperitoneally, [49]). The duration of the experiment was 29 days from CFA injection. Animals were weighed daily and clinically scored from day one to day 29 (the last day of the experiment).

### 2.5. Arthritic Assessment

In the current study, the levels of paw edema, joint diameter, foot length, and body weight were reviewed daily from day one until the end of the experiment, day 29. In this regard, the hind footpad’s paw thickness (right) was measured in each rat using a caliper. In addition, the procedure of clinical scoring was performed with the help of two independent researchers so that one was blind to the design of experiments and grouping. Furthermore, the severity of the arthritis score was examined on days 1, 7, 14, 21, and 28 and scored as follows [50,51]: 0: No erythema or swelling, 1: Slight erythema or swelling of one or more digits, 2: Swelling of the entire paw, 3: Erythema and swelling of the ankle, and 4: Ankylosis and incapacity to bend the ankle. Noteworthily, the level of severity score was the summation of the arthritis scores of the left hind limb, with a maximum of up to 4.

### 2.6. Nociceptive Behavioral Assessments

#### 2.6.1. Mechanical Allodynia

Animals were placed on an elevated box (30 cm × 30 cm × 30 cm) with a metal wire floor, and an ascending series of von Frey filaments (Bioseb^®^, Vitrolles, France) were applied in ascending style to the plantar surface. The cut-off threshold was set at 300 g to prevent tissue damage. Each filament was tested five times. A positive response was if the animal responded to at least three withdrawals out of five consecutive trials. Therefore, that gram force was considered the paw withdrawal threshold [49,50,51]. The paw withdrawal threshold was examined on days 1, 4, 8, 12, 17, 22, and 27.

#### 2.6.2. Cold Allodynia

Cold allodynia was assessed by spraying 100 μL of acetone onto the surface of the rat paw (placed over a wire mesh) without touching the skin. The response of the rat to acetone was noted for 20 s and graded on a 4-point scale as defined by Flatters and Bennett (0, no response; 1, quick withdrawal, flick, or stamp of the paw; 2, prolonged withdrawal or repeated flicking; and 3, repeated flicking of the paw with the licking of the paw). Acetone was applied three times to the hind paw, with a gap of 5 min between the acetone applications. The individual scores noted at 20 s intervals were added to obtain a single score over a cumulative period of 1 min. The minimum score was zero and the maximum possible score was nine [52]. The scores were collected on days 1, 3, 7, 13, 18, 23, and 28.

### 2.7. Thermal Hyperalgesia

The hot plate was used to assess thermal hyperalgesia. In this test, rats were individually placed on a hot plate maintained at 55 °C. The latency to the first sign of paw licking or jump response to avoid the heat nociception was taken as an index of the pain threshold; the cut-off time was set at 30 seconds to prevent any injury to the tissues of the paws [49,50,51,52,53]. The time response latency was recorded on days 1, 5, 9, 14, 19, 24, and 29.

### 2.8. Preparation of Serum and Examination of the Immunological and Biochemical Indices

On the last day of the experiment (day 29), following the deep anesthesia, 1.5 mL of blood was drawn by cardiac puncture and then collected in a 2 mL EDTA-coated microtube containing 140 µg aprotinin. Immediately, the samples were centrifuged at 2500 g, 4 °C for 10 min. Subsequently, the plasma was collected and stored at −20 °C for further evaluation. The levels of inflammatory (TNF-α, CRP, RF, and anti-CCP, MyBioSource, San Diego, CA, USA) and anti-inflammatory (IL-10 MyBioSource, San Diego, CA, USA) mediators and biochemical parameters (NO, MDA, GSH, and MDA/GSH ratio, ZellBio, Lonsee Germany) were determined by the commercially available ELISA kits according to the manufacturer’s instruction and expressed as pg per mg protein.

### 2.9. Isolation of Lymphocytes from the Spleen and Measurement of the Secretory Cytokines

Following the euthanasia, the spleen was aseptically removed and placed into a 15 mL tube containing 5 mL ice-cold RPMI-1640/FBS (RPMI-1640, 100 U/mL penicillin, 100 µg/mL streptomycin, and 2% *v*/*v* FBS). Then, the spleen was sliced and torn using the 75 µm cell strainer mesh in ice-cold RPMI-1640/FBS to provide a fine cell suspension. The obtained cell suspension was centrifuged with 450 g at 4 °C for 10 min and the supernatant was then discarded without disturbing the pellet. Subsequently, 15 mL sterilized PBS was added to the cell pellet and the suspension was gently poured onto 15 mL of Ficoll^®^ in another 50 mL sterilized conical tube and centrifuged for 20 min with 400 g at controlled room temperature (22–25 °C). The buffy coat layer was carefully removed and rinsed three times with buffer phosphate saline with 130 g for 10 min. To remove the residual red blood cells, the cells were co-cultured with 1× diluted RBC lysis buffer (10×) according to the manufacturer’s protocol and the supernatant was aspirated without disturbing the pellet. To exclude monocytes, pellets were suspended into an enriched RPMI-1640 (10% FBS, 100 U/mL penicillin, 100 µg/mL streptomycin, 2 mM L-glutamine, and 1 mM sodium pyruvate and HEPES buffer) and cultured in 250 mL flasks overnight. Afterward, the supernatant, including lymphocytes, was transferred to a 50 mL sterilized conical tube [53]. Isolated lymphocytes were cultured in RPMI-1640 enriched with 1% *v*/*v* of pen/strep (100×), 10% *v*/*v* of heat-inactivated FBS, 0.5 µg/mL amphotericin B, and 2 mM L-glutamine. Cells were maintained at 37 °c and 5% *v*/*v* CO_2_ in a humidified incubator. Eventually, the levels of IFN-γ, IL-4, IL-17, and TGF-β and the IFN-γ/IL-4 and IL-17/TGF-β ratios were measured using commercially available ELISA kits (MyBioSource, USA) according to the manufacturer’s instruction and expressed as pg/mg protein.

### 2.10. Statistical Analysis

Data were analyzed using GraphPad Prism^®^ 6 (GraphPad Software, San Diego, CA, USA) software and expressed as means ± SEM. For parametric data, a normality test was performed based on Kolmogorov–Smirnov and Bartlett’s tests to assess the homogeneity of variances. If the test was passed, comparisons between groups were made using a two-way analysis of variance (ANOVA) with the following Dunnett’s post hoc multiple comparisons test. For clinical score and body weight, repeated measures of two-way ANOVA were performed, followed by Dunnett’s multiple comparisons tests. On the other hand, non-parametric data were analyzed using Kruskal–Wallis’s test, followed by Dunn’s post hoc multiple comparisons test. *p* values (*p*) were considered statistically significant when *p* ≤ 0.05, 0.01, and 0.001. The data and statistical analysis complied with the recommendations for experimental design, analysis [54], and data sharing and presentation in preclinical pharmacology [55,56].

## 3. Results

### 3.1. Step One

The results of the measurements based on the plan of this study were performed as follows.

**Figure 1:**
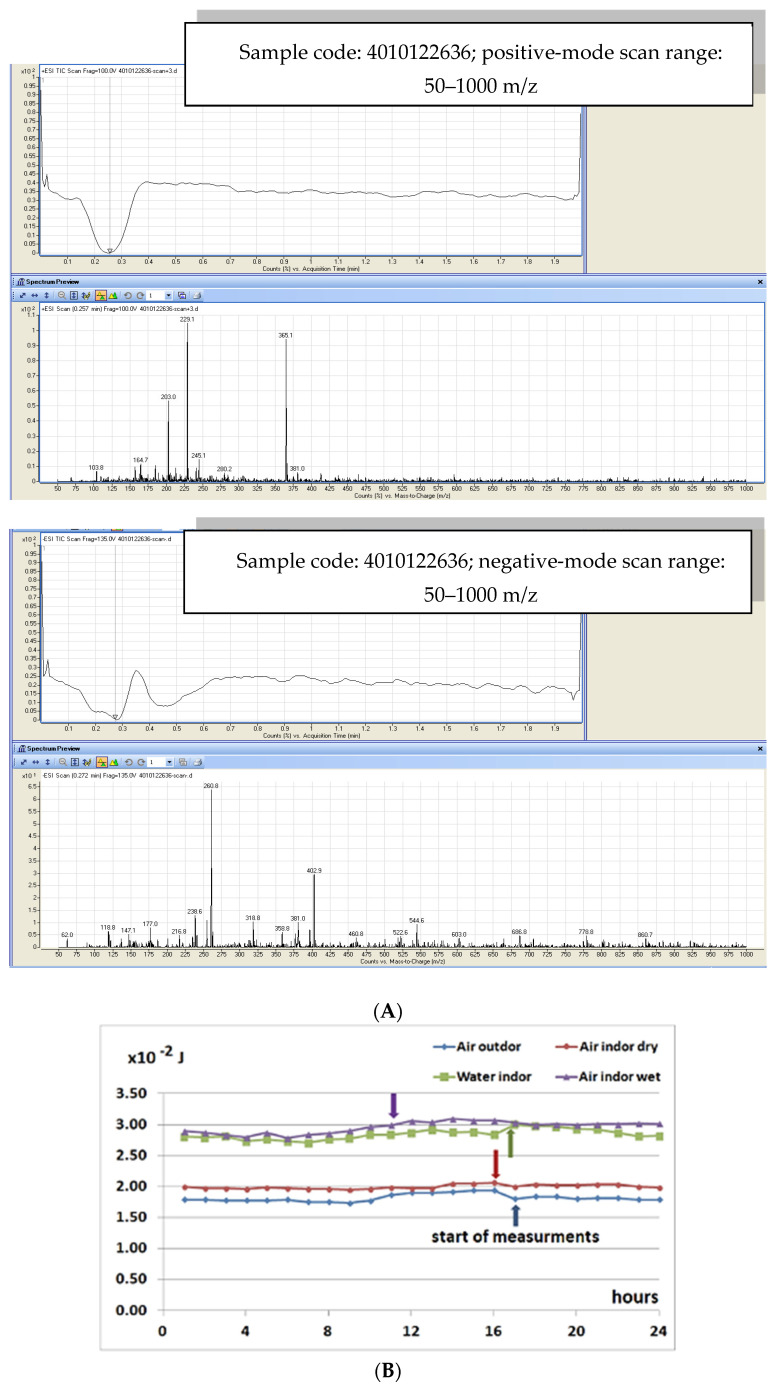

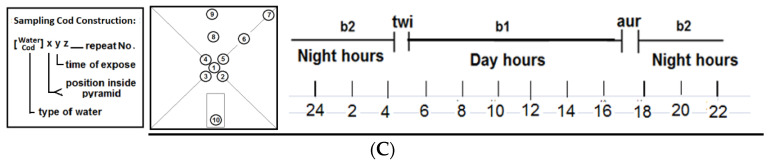
(**A**) LC/MS analysis of water enriched with vital bio-quantum information (EW). (**B**) Energy variations of air outside and inside resonator with and without water during 24 h (normalized to 4 January 2021). Arrows indicate start of measurements. (**C**) Time coding: for the duration of the twilight (twi) and aurora (aur) according to the figure, the duration between them, b1 and b2, was determined.

Continuous measurements were recorded to compare the amount of energy transferred from high-frequency bio-quantum rays to the air outside, the air inside the Fibonacci Carbon Units Absorbing Bio quantum Data (with and without water), and the water inside the Fibonacci Carbon Units Absorbing Bio quantum Data (with the water probe); continuous measurements were recorded for 24 h. From these data, the energy variations of 10 min at the beginning of each hour with 1 min intervals were extracted, and their average is shown in Figure 1.

**Figure 2:**
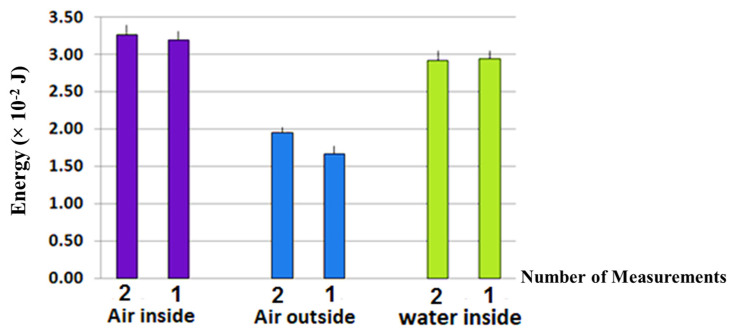
The average energy level of air outside and inside the resonator and water in the Fibonacci Carbon Unit for Bio-Quantum Data Absorption after seven days (normalized to 4 January 2021). The standard deviation of these data was 0.15 for the air outside the Fibonacci Carbon Units Absorbing Bio quantum Data and 0.08 for the air and water inside the Fibonacci Carbon Units Absorbing Bio quantum Data, which indicated the stabilization of the energy status inside the structure. The values in this diagram were normalized with the correction factor of 30 days on 20 January, considering that the time interval between the measurements lasted more than ten days and that the air measurements were performed with different cables. To check the stability of the energy levels in indoor and outdoor air and water after seven days, these measurements were re-measured with a standard cable at a time interval of 20 min.

Furthermore, Figure 1 indicates that prior to filling the unitary carbon Fibonacci data-absorbing quantum biosystems with water and throughout the entire day, the amount of energy in the air outside the unitary carbon Fibonacci data-absorbing quantum biosystem was slightly less than the air inside, with some variations that could have been due to the changes in ambient temperature at different hours of the day. After the unitary carbon Fibonacci data-absorbing quantum biosystem was filled with water, the energy transfer phenomenon to the water inside the unitary carbon Fibonacci data-absorbing quantum biosystem suddenly occurred and remained nearly unchanged at 28.4 ± 0.8 mJ during both day and night. Filling up the unitary carbon Fibonacci data-absorbing quantum biosystem has also increased the energy of the air inside it. Re-measurement of these values after seven days, shown in Figure 2, demonstrates the stability of energy levels in the air and water inside the unitary carbon Fibonacci data-absorbing quantum biosystem despite the large difference in the energy of the air outside.

The standard deviation of these data was 0.15 for the air outside the Fibonacci Carbon Units Absorbing Bio quantum Data and 0.08 for the air and water inside the Fibonacci Carbon Units Absorbing Bio quantum Data, which indicated the stabilization of the energy status inside the structure. The values in this diagram were normalized with the correction factor of 30 days on 20 January, considering that the time interval between the measurements lasted more than ten days and the air measurements were performed with different cables. To check the stability of the energy levels in indoor and outdoor air and water after seven days, these measurements were re-measured with a standard cable at a time interval of 20 min; the obtained values are shown in Figure 2.

To study the amount of energy absorbed in different types of water, volumetric flasks of 250 cc containing samples of deionized water (Dei), drinking water (Dri), distilled water (Dis), and physiological serum (Phs) were placed in similar locations (2, 3, 4, and 5) inside the resonator for 24 h with the time code “_24 h” and for 66 h with the code “_66 h”. Thereafter, the samples were measured with the following codes: “Dei 2_24 h”, “Dis 5_24 h”, “Dri 3_24 h”, and “Phs 4_24 h”. The water control samples identical to those inside the Fibonacci Carbon Units Absorbing Bio quantum Data (using location index 0) were measured with the codes Dei 0, Dri 0, Dis 0, and Phs 0, as in Table 1. The variation in the energy of these samples, when poured into plastic containers for measurements, is demonstrated in Table 1.

Moreover, Table 1 indicates that the energy of water before entering the unitary carbon Fibonacci data-absorbing quantum biosystem was 15 ± 0.1 mJ for deionized and distilled water and 16 ± 0.1 mJ for drinking water and physiological serum, showing a slight change. After 24 h, there was no noticeable change in the energy levels in the volumetric flasks’ water samples, which were measured in plastic containers. However, the standard deviation of these measurements decreased, which may have been due to the stabilization of the water energy status during this period. According to Figure 1, the direct and continuous measurements of deionized water energy on the floor of the unitary carbon Fibonacci data-absorbing quantum biosystem were taken in the range of 28 mJ. This indicated that energy was not well transferred in small-volume containers and depended on their location and size inside the unitary carbon Fibonacci data-absorbing quantum biosystem. According to Table 1, after 66 h, the deionized water energy in the volumetric flasks increased by 13 mJ; other types of water increased by 2 mJ. This confirmed the slight increase in energy transfer over time despite the energy increase limitation in small containers.

**Figure 3:**
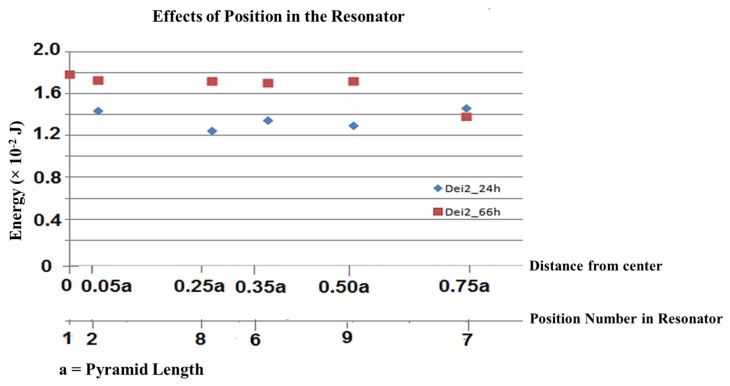
The amount of energy absorbed in different places (1, 2, 6, 7, 8, and 9) inside the Fibonacci Carbon Unit for Bio-Quantum Data Absorption, a similar water type (Dei) at two fixed durations (24 h “_24 h” and 66 h “_66 h”) were carried under experiment with the relevant coding.

To evaluate the amount of energy absorbed in different places (1, 2, 6, 7, 8, and 9) inside the Fibonacci Carbon Units Absorbing Bio quantum Data, a similar water type (Dei) at two fixed durations (24 h “_24 h” and 66 h “_66 h”) were carried under an experiment with the relevant coding. The energy changes for these samples are shown in Table 2 and the changes after 66 h are illustrated in Figure 3.

Table 2 indicates the amount of energy in the flasks located in different positions on the floor of the unitary carbon Fibonacci data-absorbing quantum biosystem. The numbers suggested that the energy alterations in the center were the maximum, and, by moving away from it in concentric circles to the side of the unitary carbon Fibonacci data-absorbing quantum biosystem, the energy decreased slightly. It is worth mentioning that the energy decreased dramatically at its corners.

In order to investigate the possibility of cumulative Bio-quantum frequency enriched water, a deionized water sample was taken from the Fibonacci Carbon Unit for Bio-Quantum Data Absorption floor water in front of the Fibonacci Carbon Unit for Bio-Quantum Data Absorption gate (Dei 10) at each of the desired times and recorded with the following coding:

After an aurora “Dei 10 aur”, After the aurora, but before twilight: “Dei 10 aur b2”, After the aurora and after the twilight: “Dei 10 aur b2 twi”, After the aurora, but before the next aurora: “Dei_24 h”; The energy changes of these samples are shown in Table 3. The results also revealed that the energy transferred from high-frequency bio-quantum rays in small flasks is cumulative and gradually increases over time, especially at certain times. However, despite this increase, the water energy level of the flasks is still lower than the water energy level of the floor of the unitary carbon Fibonacci data-absorbing quantum biosystem.

In order to evaluate the possible difference in absorption at specific moments of aurora and twilight and the time intervals between them, water samples were exposed to high-frequency bio-quantum rays for a limited duration. These measurements were taken at the Fibonacci Carbon Unit for Bio-Quantum Data Absorption gate (location 10) and with the type of deionized water from the Fibonacci Carbon Unit for Bio-Quantum Data Absorption floor (Dei 10). It should be said that the flask of the sample was filled with deionized water (Dei) and, after being placed inside the Fibonacci Carbon Units Absorbing Bio quantum data at any of the desired moments, it was emptied into a small plastic container, refilled with deionized water, placed in the same position again, and recorded with the following codes: Dei 10 twi, Dei 10 b1, Dei 10 aur, and Dei 10 b2. The energy variations of these samples are shown in Table 4.

In fact, Table 4 indicates that the energy transfer during the short period of aurora showed the highest value (15 ± 0.7 mJ), during the day held the lowest value (13.1 ± 0.5 mJ), and during the short periods of twilight and at night was the same (14 ± 0.7 mJ). In other words, the highest energy intensity of high-frequency bio-quantum rays occurred during the short periods of aurora and twilight.

The effects of the Fibonacci Carbon Unit for Bio-Quantum Data Absorption drainage in smaller containers for further measurement and glass containers inside the Fibonacci Carbon Units Absorbing Bio quantum Data were investigated using the following structure. First, the physiological serum sample was measured in a volumetric flask and then measurements were repeated after drainage into a plastic vial and glass cup. The results are shown in Table 5.

In brief, the results represented that the energy of the serum sample was 19.6 ± 0.8 mJ while it was inside the volumetric flask; after pouring it into a plastic container, it decreased slightly to 18.1 ± 0.9 mJ, and after pouring it into a glass beaker, it slightly increased to 21.1 ± 1.2 mJ (Table 5). In other words, glass or crystal containers for storing water were better than plastic containers.

Then, to investigate the effect of glass containers inside the Fibonacci Carbon Units Absorbing Bio quantum Data, the measurement related to the water’s energy level of the Fibonacci Carbon Unit for Bio-Quantum Data Absorption floor was carried out after three days when the volumetric flasks and spheres were inside the Fibonacci Carbon Units Absorbing Bio quantum Data. Furthermore, the measurements were repeated after moving all the flasks and spheres out of the Fibonacci Carbon Units Absorbing Bio quantum Data. The results are demonstrated in Table 6.

It can be concluded from Table 6 that the energy level of deionized water inside the small flask (16.9 ± 1.2 mJ) after three days was slightly less than the same water sample in a larger glass sphere container (17.9 ± 0.8 mJ) and much less than the energy level of the water at the bottom of the unitary carbon Fibonacci data-absorbing quantum biosystem (27.9 ± 0.6 mJ), where all the containers were removed. This also confirmed the results of Table 1. Furthermore, these samples were measured in small plastic containers and, therefore, had slightly lower energy than the flask and the water at the bottom of the unitary carbon Fibonacci data-absorbing quantum biosystem, which also confirmed the results of Table 5).

The results of Table 7 demonstrate that the energy of the deionized water sample inside the glass sphere container in the center of the unitary carbon Fibonacci data-absorbing quantum biosystem was 17.9 ± 0.8 mJ, which slightly changed with physical interference such as 10 h of exposure to WiFi radiation, 5 min of boiling, 9 h of freezing, and 1 min of heating in the microwave. These variations did not indicate significant changes in the amount of energy except in the microwave sample, but the increase in their standard deviation led to a decrease in the uniformity of energy levels in them.

### 3.2. Step Two

#### 3.2.1. Effects of EW and MTX and Their Combination on Body Weight in RA Rats

**Figure 4:**
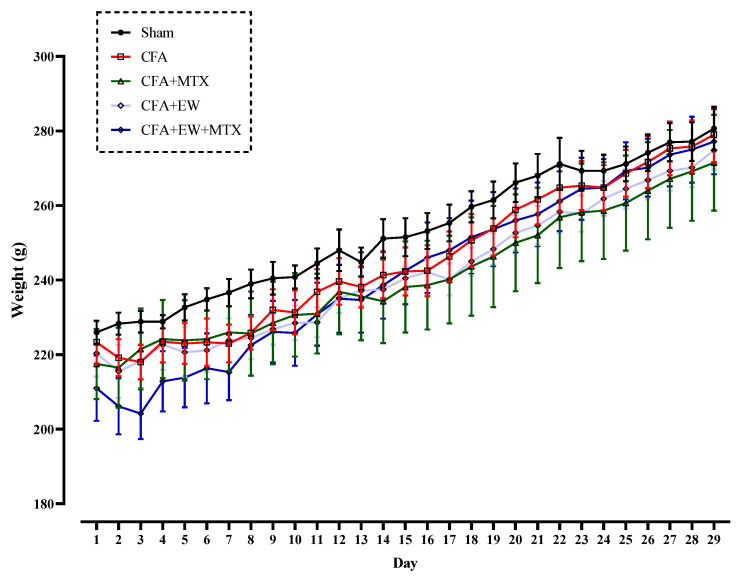
Effects of EW and MTX and their combination on body weight in RA rats. Data are expressed as mean ± SEM.

As illustrated in Figure 4, there were no significant differences in the body weight of all rats on day one of the experiment. Our results indicated that one-month medication with EW, MTX, and their combination had no significant effects on body weight compared to the sham and CFA groups (Figure 4).

#### 3.2.2. Effects of EW and MTX and Their Combination on Foot Length in RA Rats

**Figure 5:**
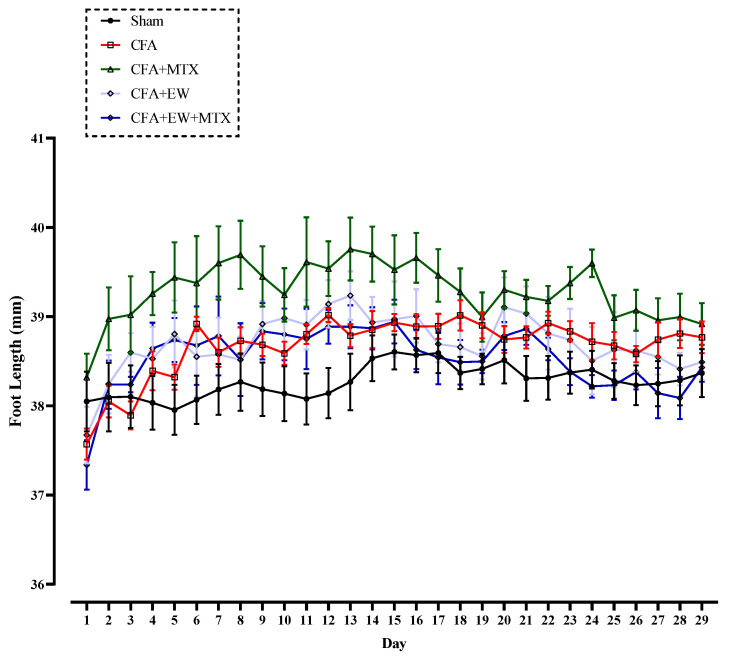
Effects of EW and MTX and their combination on foot length in RA rats. Data are expressed as mean ± SEM (n = 8).

As shown in Figure 5, there were no significant differences in foot length (mm) of all rats on day one of the experiment. Our results indicated that one-month medication with EW, MTX, and their combination did not considerably impact the foot length (mm) compared to the sham and CFA groups (Figure 5).

#### 3.2.3. Effects of EW and MTX and Their Combination on Paw Edema in RA Rats

**Figure 6:**
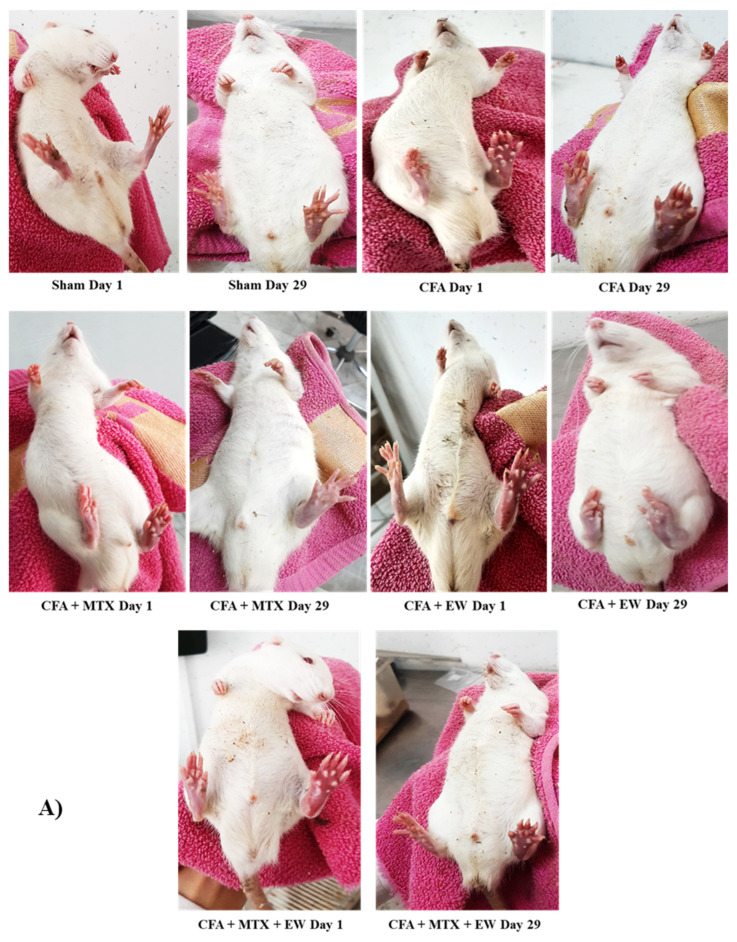

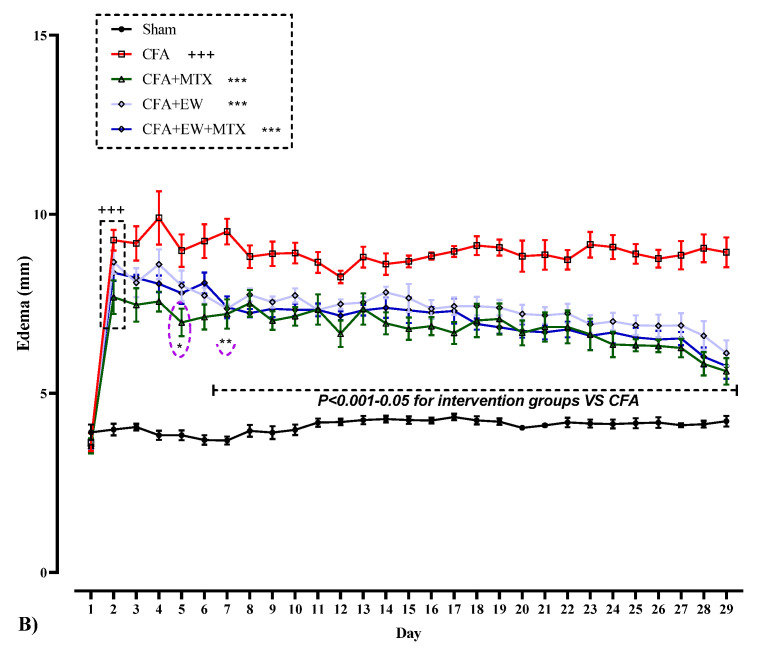
Effects of EW and MTX and their combination on paw edema in RA rats. (**A**) The figure indicates paw edema and inflammation on the first and last days of this study. (**B**) Data are expressed as mean ± SEM (n = 8). + Compared to the sham group. +++ *p* < 0.001. * Compared to the CFA group. * *p* < 0.05. ** *p* < 0.01. *** *p* < 0.001. Repeated data were compared using repeated-measurement two-way ANOVA followed by Dunnett’s multi-comparison test. Furthermore, cumulative comparisons (area under the curve) indicated in the dotted squared box were analyzed using two-way ANOVA followed by Dunnett’s multi-comparison test.

As represented in Figure 6, there were no significant differences between paw edema (mm) of all rats on day one of the experiment. However, results showed that CFA injection markedly elevated paw edema compared to the sham group (*p* < 0.001, Figure 6). In contrast, co-administration with EW, MTX, or their combinations dramatically diminished the paw edema compared to the CFA group (*p* < 0.001–0.05 for all cases, Figure 6). Noteworthily, we found that the changes were significant from the seventh day of CFA challenge.

#### 3.2.4. Effects of EW and MTX and Their Combination on Joint Diameter in RA Rats

**Figure 7:**
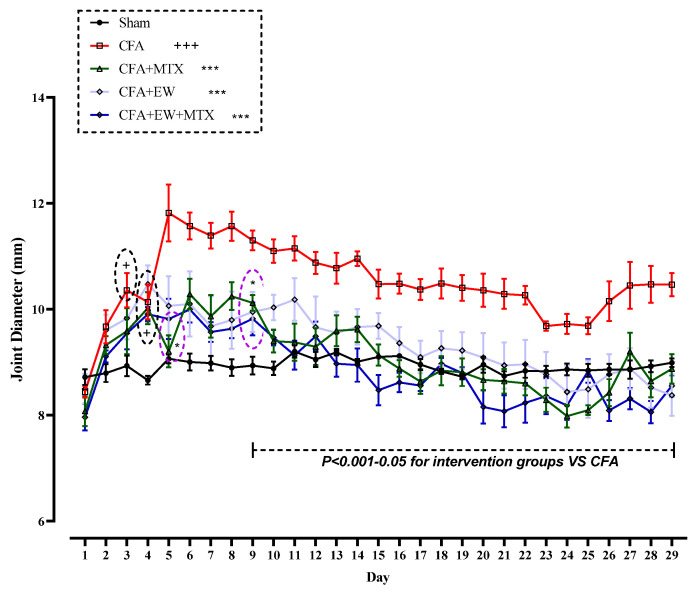
Effects of EW and MTX and their combination on joint diameter in RA rats. Data are expressed as mean ± SEM (n = 8). + Compared to the sham group. + *p* < 0.05. +++ *p* < 0.001. * Compared to the CFA group. * *p* < 0.05. *** *p* < 0.001. Repeated data were compared using repeated-measurement two-way ANOVA followed by Dunnett’s multi-comparison test. Furthermore, cumulative comparisons (area under the curve) indicated in the dotted squared box were analyzed using two-way ANOVA followed by Dunnett’s multi-comparison test.

As found in Figure 7, there were no significant differences in the joint diameter (mm) of all rats on day one of the experiment. However, the results revealed that CFA injection markedly elevated joint diameter after the third injection compared to the sham group (*p* < 0.05, Figure 7). In contrast, co-administration with EW, MTX, or their combinations notably diminished the joint diameter (mm) from the ninth day of CFA challenge compared to the CFA group (*p* < 0.001–0.05 for all cases, Figure 7).

#### 3.2.5. Effects of EW and MTX and Their Combination on Tactile Allodynia (Paw Withdrawal Threshold) in RA Rats

**Figure 8:**
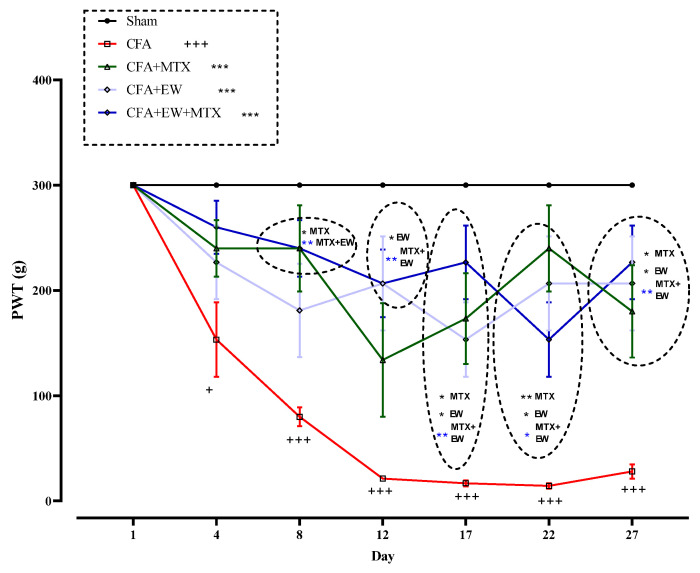
Effects of EW and MTX and their combination on tactile allodynia (paw withdrawal threshold) in RAr ats. Data are expressed as mean ± SEM (n = 8). + Compared to the sham group + *p* < 0.05. +++ *p* < 0.001. * Compared to the CFA group * *p* < 0.05. ** *p* < 0.01. *** *p* < 0.001. Repeated data were compared using repeated-measurement two-way ANOVA followed by Dunnett’s multi-comparison test. Furthermore, cumulative comparisons (area under the curve) indicated in the dotted squared box were analyzed using two-way ANOVA followed by Dunnett’s multi-comparison test.

As illustrated in Figure 8, there were no significant differences between the tactile allodynia (paw withdrawal threshold) of all rats on day one of the experiment. CFA injection meaningfully reduced the tolerance to tactile allodynia (paw withdrawal threshold) from days 4 (*p* < 0.05), 8 (*p* < 0.001), 12 (*p* < 0.001), 17 (*p* < 0.001), 22 (*p* < 0.001), and 27 (*p* < 0.001) compared to the sham group (Figure 8). On the contrary, interventions with EW, MTX (2 mg/kg/week) as a positive control, and its combination with EW significantly increased the tolerability to tactile allodynia (paw withdrawal threshold) on the fourth day after CFA injection; however, the levels of differences only were statistically significant from day eight compared to the CFA group (*p* < 0.001–0.05 for all cases, Figure 8).

#### 3.2.6. Effects of EW and MTX and Their Combination on Cold Allodynia Score in RA Rats

**Figure 9:**
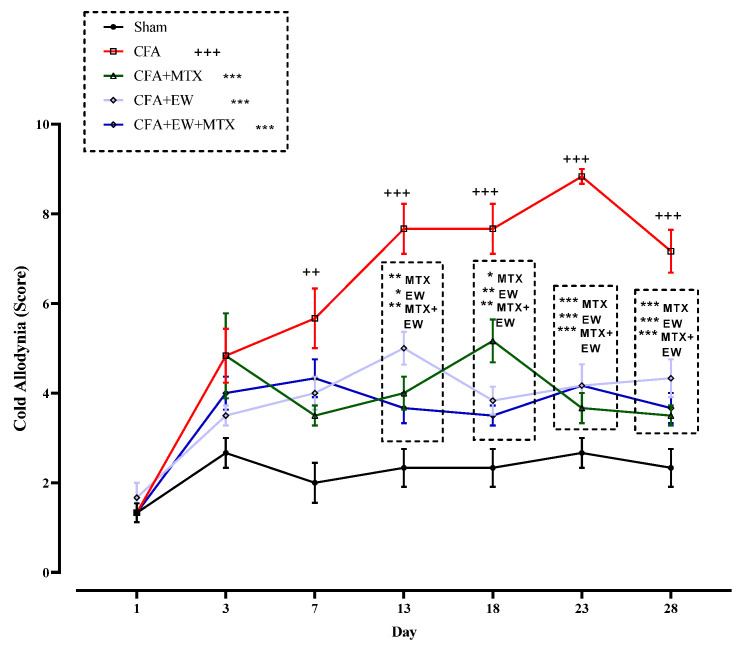
Effects of EW and MTX and their combination on cold allodynia score in RA rats. Data are expressed as mean ± SEM (n = 8). + Compared to the sham group. ++ *p* < 0.01. +++ *p* < 0.001. * Compared to the CFA group. * *p* < 0.05. ** *p* < 0.01. *** *p* < 0.001. Repeated data were compared using repeated-measurement two-way ANOVA followed by Dunnett’s multi-comparison test. Furthermore, cumulative comparisons (area under the curve) indicated in the dotted squared box were analyzed using two-way ANOVA followed by Dunnett’s multi-comparison test.

As illustrated in Figure 9, no significant differences existed between the cold allodynia scores of all rats on day one of the experiment. However, CFA injection meaningfully enhanced the score of cold allodynia from days 7 (*p* < 0.01), 13 (*p* < 0.001), 18 (*p* < 0.001), 23 (*p* < 0.001), and 28 (*p* < 0.001) compared to the sham group (Figure 9). On the contrary, utilizing EW, MTX (2 mg/kg/week) as a positive control, and its combination with EW considerably attenuated the cold allodynia score on the 13th day after CFA injection compared to the CFA group (*p* < 0.001–0.05 for all cases, Figure 9).

#### 3.2.7. Effects of EW and MTX and Their Combination on Thermal Hyperalgesia (Time Response Latency, Sec) in RA Rats

**Figure 10:**
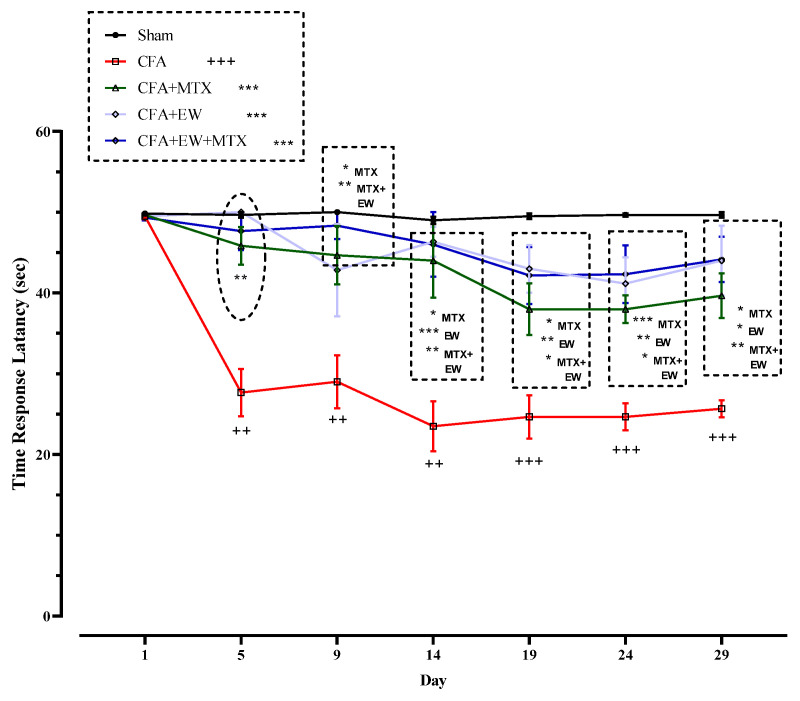
Effects of EW and MTX and their combination on thermal hyperalgesia (time response latency, sec) in RA rats. Data are expressed as mean ± SEM (n = 8). + Compared to the sham group. ++ *p* < 0.01. +++ *p* < 0.001. * Compared to the CFA group. * *p* < 0.05. ** *p* < 0.01. *** *p* < 0.001. Repeated data were compared using repeated-measurement two-way ANOVA followed by Dunnett’s multi-comparison test. Furthermore, cumulative comparisons (area under the curve) indicated in the dotted squared box were analyzed using two-way ANOVA followed by Dunnett’s multi-comparison test.

As demonstrated in Figure 10, there were no significant differences between all rats’ thermal hyperalgesia (time response latency, sec) on day one of the experiment. However, CFA injection meaningfully diminished the time response latency from days 5 (*p* < 0.01), 9 (*p* < 0.01), 14 (*p* < 0.01), 19 (*p* < 0.001), 24 (*p* < 0.001), and 29 (*p* < 0.001) compared to the sham group (Figure 10). On the other hand, co-administrating EW, MTX (2 mg/kg/week) as a positive control, and its combination with EW considerably propagated the time response latency on the fifth day after CFA injection compared to the CFA group (*p* < 0.001–0.05 for all cases, Figure 10).

#### 3.2.8. Effects of EW and MTX and Their Combination on Arthritis Score in RA Rats

**Figure 11:**
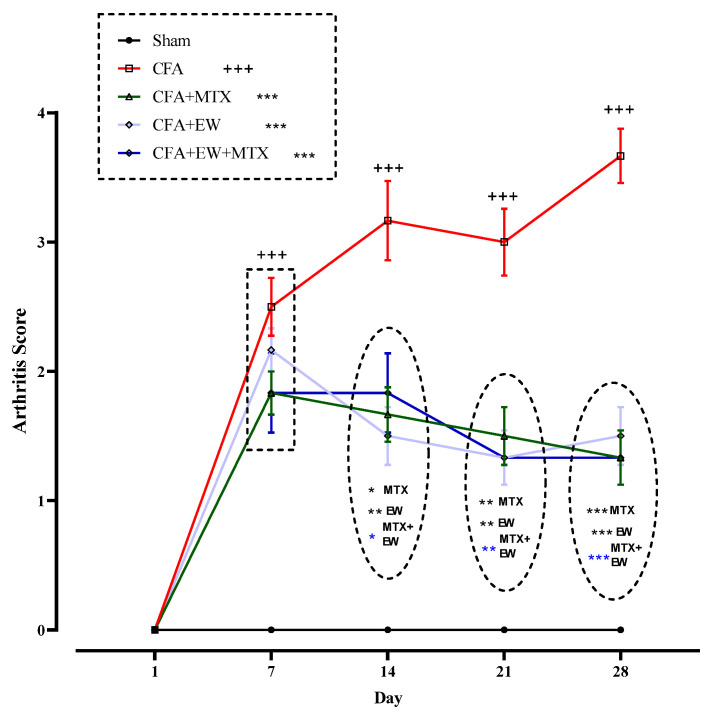
Effects of EW and MTX and their combination on arthritis score in RA rats. Data are expressed as mean ± SEM (n = 8). + Compared to the sham group. +++ *p* < 0.001. * Compared to the CFA group. * *p* < 0.05. ** *p* < 0.01. *** *p* < 0.001. Repeated data were compared using repeated-measurement two-way ANOVA followed by Dunnett’s multi-comparison test. Furthermore, cumulative comparisons (area under the curve) indicated in the dotted squared box were analyzed using two-way ANOVA followed by Dunnett’s multi-comparison test.

As demonstrated in Figure 11, there were no significant differences between the arthritis scores of all rats on day one of the experiment. Arthritis score was significantly increased following the CFA injection from days 7 (*p* < 0.001), 14 (*p* < 0.001), 21 (*p* < 0.001), and 28 (*p* < 0.001) compared to the sham group (Figure 11). Interestingly, treatment with EW, MTX (2 mg/kg/week) as a positive control, and its combination with EW considerably diminished the arthritis score on the 14th day after CFA injection compared to the CFA group (*p* < 0.001–0.05 for all cases, Figure 11).

#### 3.2.9. Effects of EW and MTX and Their Combination on the Serum Levels of Inflammatory and Anti-Inflammatory Mediators in RA Rats

**Figure 12:**
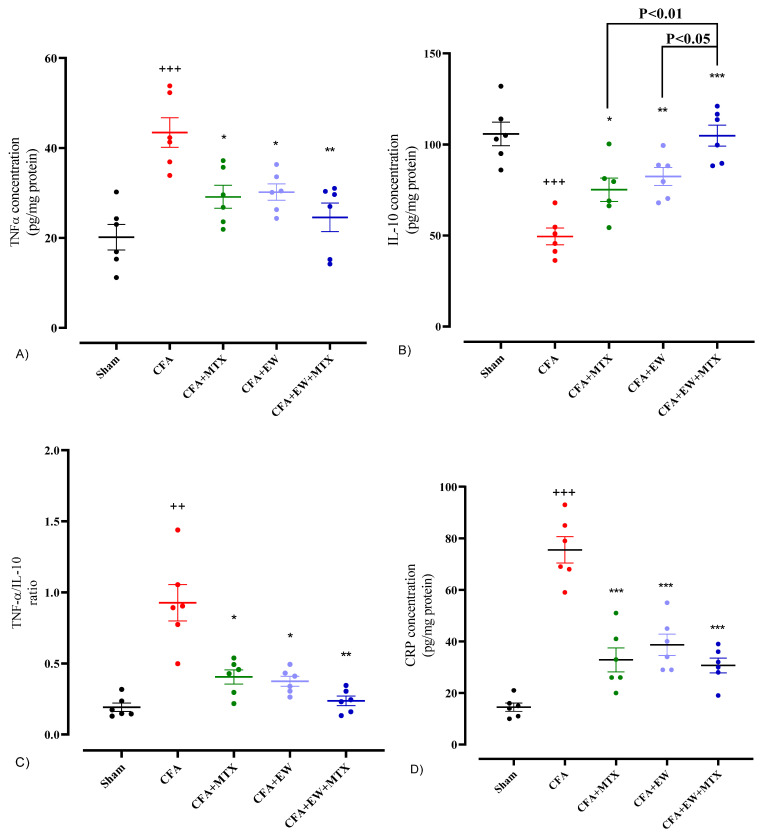

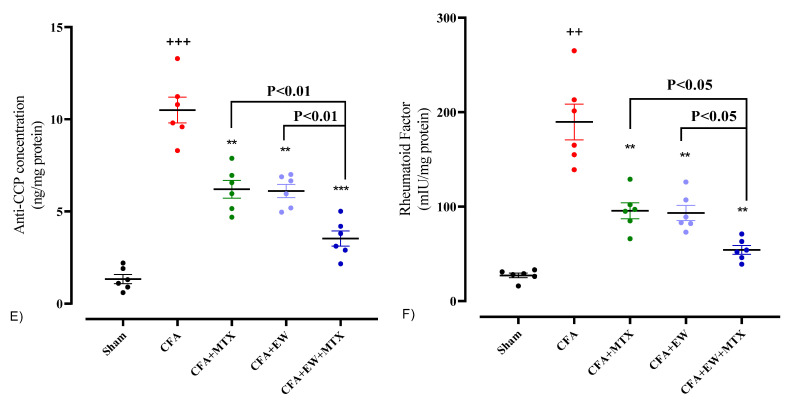
Effects of EW and MTX and their combination on serum levels of (**A**) TNF-α, (**B**) IL-10, (**C**) the TNF-α/IL-10 ratio, (**D**) CRP, (**E**) anti-CCP, and (**F**) rheumatoid factor in RA rats. Data were expressed as mean ± SEM (n = 6). + Compared to the sham group. ++ *p* < 0.01. +++ *p* < 0.001. * Compared to the CFA group. * *p* < 0.05. ** *p* < 0.01. *** *p* < 0.001. Data were analyzed and compared using one-way ANOVA followed by Dunnet-T3’s multi-comparison test.

The results of the current study indicated that CFA-induced RA led to a significant increase in the serum levels of inflammatory mediators, including TNF-α (*p* < 0.001, Figure 12A), the TNF-α/IL-10 ratio (*p* < 0.01, Figure 12C), CRP (*p* < 0.001, Figure 12D), anti-CCP (*p* < 0.001, Figure 12E), and RF (*p* < 0.01, Figure 12F), and a marked reduction in the serum levels of anti-inflammatory cytokine IL-10 (*p* < 0.001, Figure 12B) compared to the sham group. As a positive control group, MTX (2 mg/kg/week) meaningfully reversed the serum levels of TNF-α (*p* < 0.05, Figure 12A), the TNF-α/IL-10 ratio (*p* < 0.05, Figure 12C), CRP (*p* < 0.001, Figure 12D), anti-CCP (*p* < 0.01, Figure 12E), and RF (*p* < 0.01, Figure 12F) and also improved IL-10 (*p* < 0.05, Figure 12B) in comparison to the CFA group. Furthermore, we observed that daily administration of EW alone or in combination with MTX (2 mg/kg/week) caused an impressive reduction in the serum levels of TNF-α (*p* < 0.05 and *p* < 0.01, respectively, Figure 12A), the TNF-α/IL-10 ratio (*p* < 0.05 and *p* < 0.01, respectively, Figure 12C), CRP (*p* < 0.001 for both cases, Figure 12D), anti-CCP (*p* < 0.01 and *p* < 0.001, respectively, Figure 12E), and RF (*p* < 0.01 for both cases, Figure 12F) and also strikingly improved IL-10 (*p* < 0.01 and *p* < 0.001, respectively, Figure 12B) in comparison to the CFA group.

#### 3.2.10. Effects of EW and MTX and Their Combination on Serum Levels of Oxidative Stress Markers in RA Rats

**Figure 13:**
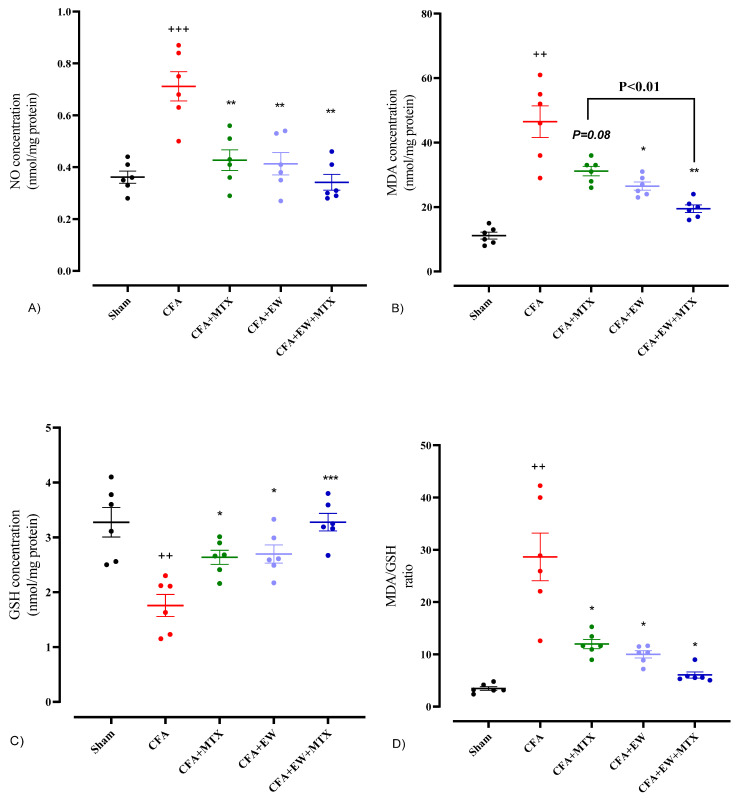
Effects of EW and MTX and their combination on the serum levels of (**A**) NO, (**B**) MDA, (**C**) GSH, and (**D**) MDA/GSH ratio in RA rats. Data are expressed as mean ± SEM (n = 6). + Compared to the sham group. ++ *p* < 0.01. +++ *p* < 0.001. * Compared to the CFA group. * *p* < 0.05. ** *p* < 0.01. *** *p* < 0.001. Data were analyzed and compared using one-way ANOVA followed by Dunnet-T3’s multi-comparison test.

As indicated in Figure 13, CFA-injected rats had more substantial serum levels of NO (*p* < 0.001, Figure 13A), MDA (*p* < 0.01, Figure 13B), and MDA/GSH ratio (*p* < 0.01, Figure 13D) and lower levels of GSH (*p* < 0.01, Figure 13C) compared to the sham group. In contrast, the results revealed that treatment with MTX (2 mg/kg/week), daily administration of EW alone, or its combination with MTX (2 mg/kg/week) resulted in an apparent restoration in the serum levels of NO (*p* < 0.01 for all cases, Figure 13A), MDA (*p* < 0.08, *p* < 0.05, and *p* < 0.01, respectively, Figure 13B), GSH (*p* < 0.05, *p* < 0.05, and *p* < 0.001, respectively, Figure 13C), and MDA/GSH ratio (*p* < 0.01 for all cases, Figure 13D) compared to the CFA group.

#### 3.2.11. Effects of EW and MTX and Their Combination on Lymphocytes Secretory Cytokines in RA Rats

**Figure 14:**
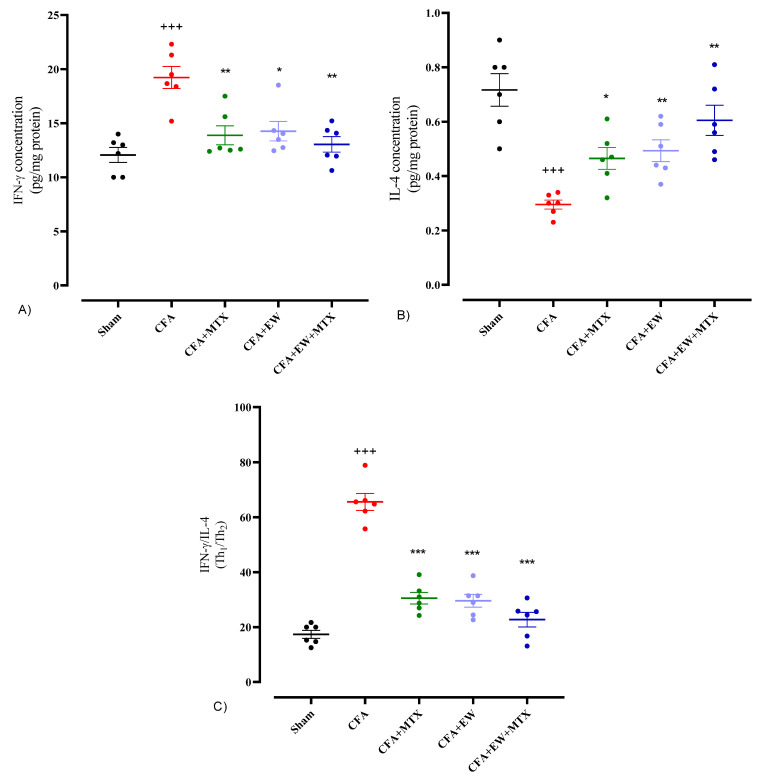
Effects of EW and MTX and their combination on lymphocytes secretory cytokines levels of (**A**) IFN-γ, (**B**) IL-4, and (**C**) IFN-γ/IL-4 ratio in RA rats. Data are expressed as mean ± SEM (n = 6). + Compared to the sham group. +++ *p* < 0.001. * Compared to the CFA group. * *p* < 0.05. ** *p* < 0.01. *** *p* < 0.001. Data were analyzed and compared using one-way ANOVA followed by Dunnet-T3’s multi-comparison test.

**Figure 15:**
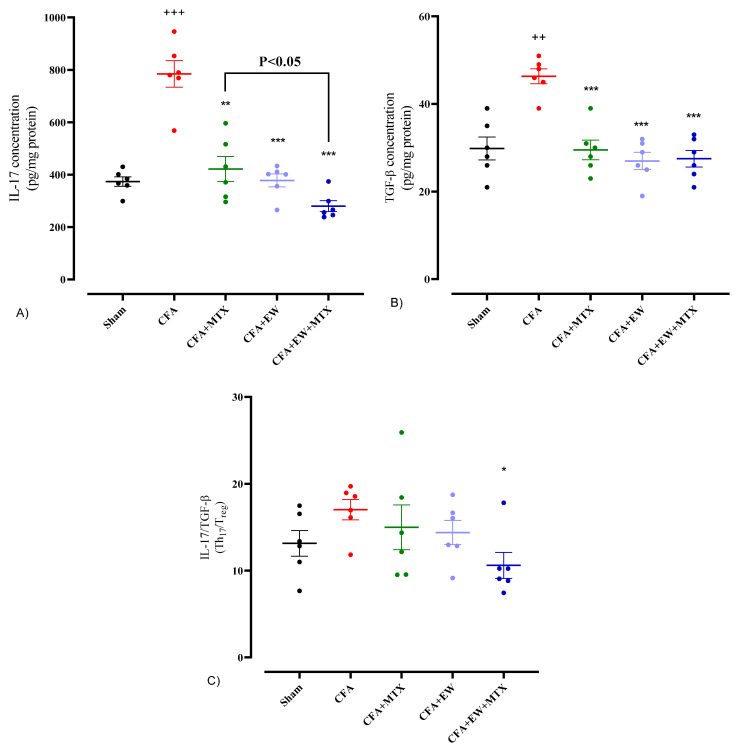
Effects of EW and MTX and their combination on lymphocytes secretory cytokines levels of (**A**) IL-17, (**B**) TGF-β, and (**C**) IL-17/TGF-β ratio in RA rats. Data are expressed as mean ± SEM (n = 6). + Compared to the sham group. ++ *p* < 0.01. +++ *p* < 0.001. * Compared to the CFA group. * *p* < 0.05. ** *p* < 0.01. ***. *p* < 0.001. Data were analyzed and compared using one-way ANOVA followed by Dunnet-T3’s multi-comparison test.

Experimentally, CFA-induced RA caused a significant elevation in lymphocyte secretory cytokines levels, including IFN-γ (*p* < 0.001, Figure 14A), the IFN-γ/IL-4 ratio (*p* < 0.001, Figure 14C), IL-17 (*p* < 0.001, Figure 15A), and TGF-β (*p* < 0.01, Figure 15B), and a crucial attenuation in the levels of IL-4 (*p* < 0.001, Figure 14B) compared to the sham group. Interestingly, interventions with MTX (2 mg/kg/week), daily administration of EW alone, or its combination with MTX (2 mg/kg/week) resulted in a glaring restoration in the levels of IFN-γ (*p* < 0.01, *p* < 0.05, and *p* < 0.01, respectively, Figure 14A), IL-4 (*p* < 0.05, *p* < 0.01, and *p* < 0.01, respectively, Figure 14B), IFN-γ/IL-4 ratio (*p* < 0.001 for all cases, Figure 14C), IL-17 (*p* < 0.01, *p* < 0.001, and *p* < 0.001, respectively, Figure 15A), and TGF-β (*p* < 0.001 for all cases, Figure 15B) compared to the CFA group. Furthermore, we observed that the IL-17/TGF-β ratio had no significant difference between the CFA-treatment-alone and sham groups, although the combination of MTX and EW significantly reduced these levels compared to the CFA-treated group (*p* < 0.05, Figure 15C).

## 4. Discussion

To the best of our knowledge, this was the first study determining the potential anti-arthritic properties of EW on a CFA-induced experimental arthritis model in rats. We found that EW remarkably ameliorated thermal hyperalgesia, cold allodynia, and tactile allodynia results. In addition, EW also notably attenuated arthritis score, joint diameter, inflammatory cytokines, and oxidative markers while propagating anti-inflammatory and anti-oxidative mediators.

CFA is experimentally used to induce arthritis in animals that mimics the human RA features, including hypersensitivity to mechanical and heat stimuli, joint and bone destruction, and joint inflammation [57]. The present study evaluated paw edema and joint diameter as the first parameters for RA in rats. In addition, we measured arthritis scores indicating paw erythema or swelling in rats [58]. Our results showed that CFA injection meaningfully stimulated paw edema, joint diameter, and arthritis score in rats. In line with our results, previous studies showed that CFA enhanced paw edema, thickness, volume, and arthritis score [59,60]. In contrast, treatment with MTX, EW, and their combination notably alleviated paw edema, joint diameter, and arthritis score following CFA-induced RA. The decrement in arthritis score and paw volume following treatment with EW may have originated from the suppression of inflammatory markers, resulting in diminishing arthritis severity [61].

MTX is considered the most commonly used and effective DMARD in treating RA [62]. It has been emphasized that the regular use of MTX is accompanied by increased liver enzymes and hepatotoxicity [63]. Furthermore, it has been suggested that the mechanism responsible for the hepatotoxicity of MTX is oxidative stress and inflammation [64]. Therefore, investigating new anti-RA agents with clinical efficacy and fewer adverse effects is crucial. In addition, identifying new anti-oxidative and anti-inflammatory therapeutics that potentiate MTX’s anti-RA effects and inhibit its adverse effects is beneficial [65]. Therefore, we investigated the anti-RA properties of EW alone and in combination with MTX in the RA rat model. Our results revealed that treatment with MTX or EW strikingly ameliorated arthritis score, oxidative stress, and inflammation in CFA-induced RA in rats.

Interestingly, the combined treatment of MTX and EW showed more potent anti-RA effects than each one alone. Similarly, Li and coworkers showed that CFA increased paw volume, swelling, and inflammation in X-ray examinations in rats. However, treatment with MTX markedly reduced paw volume and joint swelling following CFA-induced RA in rats. They also noticed that combined treatment of MTX with andrographolide, a natural anti-oxidant and anti-inflammatory agent, showed more substantial anti-RA effects and hepato-protective properties [49].

Several pieces of evidence suggest that CFA leads to inflammatory pain in animals. It may sensitize pain neurons due to the stimulation of inflammatory cytokines, resulting in diminishing pain thresholds [66]. Therefore, we performed thermal hyperalgesia, cold allodynia, and tactile allodynia tests in our study. We found that CFA injection meaningfully attenuated the time response latency in thermal hyperalgesia and the tolerability to tactile allodynia while promoting a cold allodynia score. In contrast, treatment with MTX, EW, or their combination notably ameliorated the results of thermal hyperalgesia, cold allodynia, and tactile allodynia tests following CFA-induction of RA in rats. In accordance with our results, Zhao et al. supported that catalpol, a natural anti-oxidant and anti-inflammatory glycoside, significantly mitigated CFA-induced mechanical allodynia and thermal hyperalgesia in rats [67]. Another study also reported that astaxanthin ameliorated the results of mechanical allodynia, mechanical hyperalgesia, cold allodynia, and thermal hyperalgesia in CFA-induced RA in rats [50]. It may be suggested that treatment with MTX, EW, or their combination significantly restores the pain threshold in RA rats.

In accordance with our results, Ma and coworkers conducted a meta-analysis on 16 randomized controlled trials investigating 1300 patients with osteoarthritis. They supported the idea that thermal mineral waters therapy notably diminished pain, as evaluated by the visual analog scale (VAS) and Western Ontario and McMaster Universities (WOMAC) scores. It also improved joint function and quality of life, as assessed by the European Quality of Life 5-dimension scale (EQ-5D) and health assessment questionnaire (HAQ) in patients with osteoarthritis [68]. Similarly, cold water immersion with a temperature between 11 and 15 °C showed promising effects in managing muscle soreness [69]. Kulisch et al. also reported that thermal water markedly attenuated pain (VAS score) while improving the motion of the lumbar spine and quality of life compared to tap water in patients with chronic low back pain [70]. These studies may confirm the therapeutic effects of different types of water and align with our results regarding the anti-RA properties of EW.

Oxidative stress plays an essential role in RA pathophysiology in animals and humans [71]. Indeed, the imbalance between the levels of oxidant and anti-oxidant markers leads to oxidative stress and also inflammatory response. Studies in RA patients revealed the stimulated ROS generation, lipid peroxidation, and DNA damage while mitigating the activities of anti-oxidant enzymes [72,73]. The present study found that CFA notably increased oxidative stress while suppressing anti-oxidative markers in rats. Therefore, anti-oxidants may be a promising treatment strategy for RA patients. In this regard, we evaluated the levels of oxidant and anti-oxidant markers in CFA-induced RA rats. Our results showed that treatment with MTX, EW, or their combination remarkably decreased oxidative markers (NO and MDA levels and MDA/GSH ratio) while elevating anti-oxidant status (GSH levels) in CFA-induced RA in rats. Previous studies also show that natural anti-oxidative compounds such as fustin and astaxanthin firmly diminish MDA and ROS levels while enhancing GSH levels and SOD and catalase activities in CFA-induced RA in rats [50,59]. Taken together, EW possesses potent anti-oxidative effects against CFA-induced RA.

Serum CRP levels are extensively used as a clinical marker of inflammation. CRP expression is induced by inflammatory cytokines, including IL-6, TNF-α, and IL-1β [74]. In addition, anti-CCP antibodies and rheumatoid factors are also known as valuable markers in RA patients [75]. It has been supported that CRP, anti-CCP, and rheumatoid factor levels are significantly associated with disease activity and chronicity, histological changes in the synovium, radiological progression, and the penetration of inflammatory cytokines into the synovium [76]. Therefore, we measured CRP, anti-CCP, and rheumatoid factor levels in our study. We found that CFA notably elevated the levels of these markers in rats. In contrast, treatment with MTX, EW, or their combination markedly decreased CRP, anti-CCP, and rheumatoid factor levels in CFA-induced-RA rats. Collectively, EW may possess promising anti-RA and anti-inflammatory effects.

Several pieces of evidence emphasize that chronic inflammation plays a critical role in the pathogenesis of RA. In fact, stimulated levels of inflammatory cytokines, namely, TNF-α and IL-6, while suppressed levels of anti-inflammatory cytokines, including IL-10, occur in RA [77]. Stimulating inflammatory cytokines leads to synovial inflammation and the destruction of cartilage and bone. In this regard, inhibitors of TNF-α, such as Etanercept, Infliximab, and Adalimumab, are considered new strategies for RA treatment. However, these drugs should be used in injection form and may cause serious side effects, including infections, malignancies, and congestive heart failure [78]. The present study showed that CFA remarkably propagated TNF-α levels while diminishing IL-10 in rats. Additionally, treatment with MTX, EW, or their combination provided a significant decrement in TNF-α levels while elevating IL-10 levels following CFA-induction of RA in rats. These results may confirm the anti-inflammatory and anti-RA properties of EW.

The mycobacterial components in the CFA stimulate vigorous immune regulation following intraplantar injection in rat paws. Indeed, the imbalance between T helper (Th) 1 and Th2 cells is associated with the chronic and robust immune response in patients with RA. CFA causes the stimulation of Th1 cells along with suppressed Th2 cell differentiation. Th1 cells are linked with the secretion of inflammatory cytokines, including IFN-γ and IL-2, leading to cartilage destruction, bone erosion, synovitis, the prevention of osteoblast differentiation, and the propagation of osteoclast formation. In contrast, Th2 cells produce anti-inflammatory cytokines, such as IL-4 and IL-13, inhibiting bone resorption and the secretion of inflammatory cytokines [79,80]. Therefore, in the present study, we measured IFN-γ and IL-4 as Th1- and Th2-secreted cytokines, respectively. We showed that CFA meaningfully promoted the IFN-γ levels and IFN-γ/IL-4 ratio while alleviating IL-4 levels. In contrast, treatment with MTX, EW, or their combination remarkably suppressed IFN-γ levels and the IFN-γ/IL-4 ratio while elevating IL-4 levels in CFA-induced-RA rats. These results may prove the immunomodulatory effects of EW through regulating Th1/Th2 balance.

Furthermore, the imbalance between Th17 and regulatory T cells (Tregs) is also involved in the formation of RA. Th17 cells secrete IL-17 and IL-21, which cause bone erosion and stimulate the production of pro-inflammatory cytokines. Moreover, Tregs produce TGF-β, associated with immune regulation [79,81]. Therefore, in the present study, we measured IL-17 and TGF-β as Th17- and Tregs-produced markers, respectively. Our results revealed that CFA injection markedly propagated IL-17 and TGF-β levels and the IL-17/TGF-β ratio in rats. However, treatment with MTX and EW significantly decreased IL-17 and TGF-β levels following CFA-induction of RA in rats. Additionally, only the combination therapy of MTX and EW notably attenuated the IL-17/TGF-β ratio. Collectively, EW may possess immunomodulatory effects by modulating Th17/Tregs balance.

One of the limitations of the current study was that we were not able to evaluate any molecular mechanism. Therefore, we suggest performing further in-depth molecular investigations in lymphocytes and further animal models to elucidate what molecular mechanisms could be modified with “EW water” treatment.

## 5. Conclusions

We revealed that EW possesses anti-arthritic effects, possibly through anti-oxidative, anti-inflammatory, and immunomodulatory properties, in an animal model (rats). Again, it is worth mentioning that we performed the current study in an animal model of RA induced by CFA and could not completely mimic the entire and complex pathophysiology of rheumatoid arthritis, especially with a very short intervention time (29 days of investigation). Collectively, EW may be a promising agent to help relieve the pain, allodynia, and inflammation associated with RA and constitute support in the symptomatic control of arthritis, especially that associated with methotrexate treatment. However, further clinical studies are necessary to understand the anti-RA effects of EW better.

## Figures and Tables

**Table 1:** Transferred energy from high-frequency bio-quantum rays to water before and after 44 and 66 h in the resonator.

Type of Water	Before Filling in the Resonator		After 24 h in the Resonator (24 h) ^®^		After 66 h in the Resonator (66 h) ^©^	
	Sampling Code	Mean ± SD mJ	Sampling Code	Mean ± SD mJ	Sampling Code	Mean ± SD mJ
Deionized	Dei 0	14.7 ± 1.4	Dei 2_24 h	14.5 ± 0.9	Dei 2_66 h	27.9 ± 0.7
Distilled	Dis 0	15.0 ± 1.2	Dis 5_24 h	15.2 ± 0.8	Dis 5_66 h	17.6 ± 0.8
Drinking	Dri 0	16.7 ± 1.4	Dri 3_24 h	17. ± 0.5	Dri 3_66 h	19.2 ± 1.0
Physiological serum	Phs 0	16.2 ± 0.9	Phs 4_24 h	16.1 ± 0.5	Phs 4_66 h	18.0 ± 0.8

^®^—with flasks and water probe in resonator; ^©^—with flasks, water sphere, and air sensor in resonator for next 44 h.

**Table 2:** The amount of energy absorbed in different places inside the Fibonacci Carbon Unit for Bio-Quantum Data Absorption.

Position No.	Distance from Center	Sampling Code	Mean ± SD mJ	Sampling Code	Mean ± SD mJ
1	0			Dei 1_66 h	17.9 ± 0.8
2	0.12a	Dei 2_24 h	14.5 ± 0.9	Dei 2_66 h	17.4 ± 1.4
8	0.25a	Dei 8_24 h	12.6 ± 0.5	Dei 8_66 h	17.3 ± 0.8
6	0.35a	Dei 6_24 h	13.6 ± 0.7	Dei 6_66 h	17.1 ± 0.8
9	0.50a	Dei 9_24 h	14.7 ± 0.6	Dei 9_66 h	17.3 ± 0.8
7	0.70a	Dei 7_24 h	13.1 ± 0.7	Dei 7_66 h	13.9 ± 0.7

Deionized water = Dei, a = Pyramidal length.

**Table 3:** Cumulative effect of Bio-quantum frequency enriched water.

Time of Cosmic Exposure	Exposure Duration	Sampling Code	Mean ± SD mJ
Aurora	20 min	Dei 10 aur	13.8 ± 0.6
Aurora tills before twilight	12 h	Dei 10 aur b2	14.5 ± 0.6
Aurora tills after twilight	12:20	Dei 10 aur b2 twi	14.9 ± 0.6
Aurora tills next aurora	24 h	Dei_24 h	15.12 ± 0.7

After an aurora “Dei 10 aur”. After the aurora, but before twilight: “Dei 10 aur b2”. After the aurora and after the twilight: “Dei 10 aur b2 twi”. After the aurora, but before the next aurora: “Dei_24 h”.

**Table 4:** Radiation absorption at specific moments.

Time	Exposure Duration	Sampling Code	Mean ± SD mJ
Twilight	20 min	Dei 10 twi	15.0 ± 0.7
Between twi and aur	11:40 h	Dei 10 b1	13.1 ± 0.5
Aurora	20 min	Dei 10 aur	14.1 ± 0.7
Between aur and tei	11:40 h	Dei 10 b2	14.3 ± 0.7

After aurora: “Dei 10 aur”. After aurora but before twilight: “Dei 10 aur b2”. After aurora and after twilight: “Dei 10 aur b2 twi”. After aurora but before the next aurora: “Dei_24 h”.

**Table 5:** Effect of using different vial types.

Measuring Containers	Sampling Code	Mean ± SD mJ
Flask	phs 4_66 h	19.6 ± 0.8
Plastic vial	phs 4_66 h	18.1 ± 0.9
Glass cup	phs 4_66 h	21.1 ± 1.2

**Table 6:** Effect of glass containers inside the resonator.

The Water of the Fibonacci Carbon Unit for Bio-Quantum Data Absorption Floor	Sampling Code	Mean ± SD mJ
In flask (flasks were inside the Fibonacci Carbon Unit for Bio-Quantum Data Absorption)	Dei 10_66 h	16.9 ± 1.2
In sphere (flasks and spheres were inside the Fibonacci Carbon Unit for Bio-Quantum Data Absorption)	Dei 1_66 h	17.9 ± 0.8
Fibonacci Carbon Unit for Bio-Quantum Data Absorption floor (without flasks and spheres inside Fibonacci Carbon Unit for Bio-Quantum Data Absorption)	Dei 10_66 h	27.9 ± 0.6

Measurements were made on plastic vials.

**Table 7:** Effect of physical changes in energy stored in water Dei 1_66.

Physical Changes	Mean ± SD mJ
10 h of exposure to WiFi	17.8 ± 2.0
5 min of boiling	17.5 ± 1.9
9 h of freezing	18.3 ± 1.8
1 min of heating in the microwave	16.1 ± 1.0

## Data Availability

The original contributions presented in this study are included in the article/Supplementary Materials. Further inquiries can be directed to the corresponding author(s).

## Supplementary Materials

The following supporting information can be downloaded at https://www.mdpi.com/article/10.3390/brainsci15030232/s1.
