# Supplementary material for: Quantum Health Accelerator® Ameliorates CFA-Induced Animal Model of Rheumatoid Arthritis: Investigating the Role of Immunomodulatory and Anti-Oxidative Effects
Source: Brain Sci. 2025 Feb 23;15(3):232. doi: 10.3390/brainsci15030232 (PMC11940038; doi:10.3390/brainsci15030232)
Supplement: Supplementary file 1 [file brainsci-15-00232-s001.zip › brainsci-3499389-supplementary for conversion.pdf]

BEHIND OUR CLAIMS (Technical Appendix of the Article)

In quantum biology, quantum information refers to the structures and quantum states within cells and biological molecules that are continuously exchanged with their surrounding environment. This process involves vibrations, quantum waves, and even quantum correlations that are essential for maintaining homeostasis and supporting the development of life.

One of the fundamental concepts of this approach is that life cannot be fully explained by classical mechanics alone. In fact, this theory posits that quantum processes such as superposition, entanglement, and quantum tunneling are involved not only at the molecular level but also within more complex systems like the brain and immune system. This implies a form of informational linkage among all components of a living system, interacting with each other through a network of quantum information.

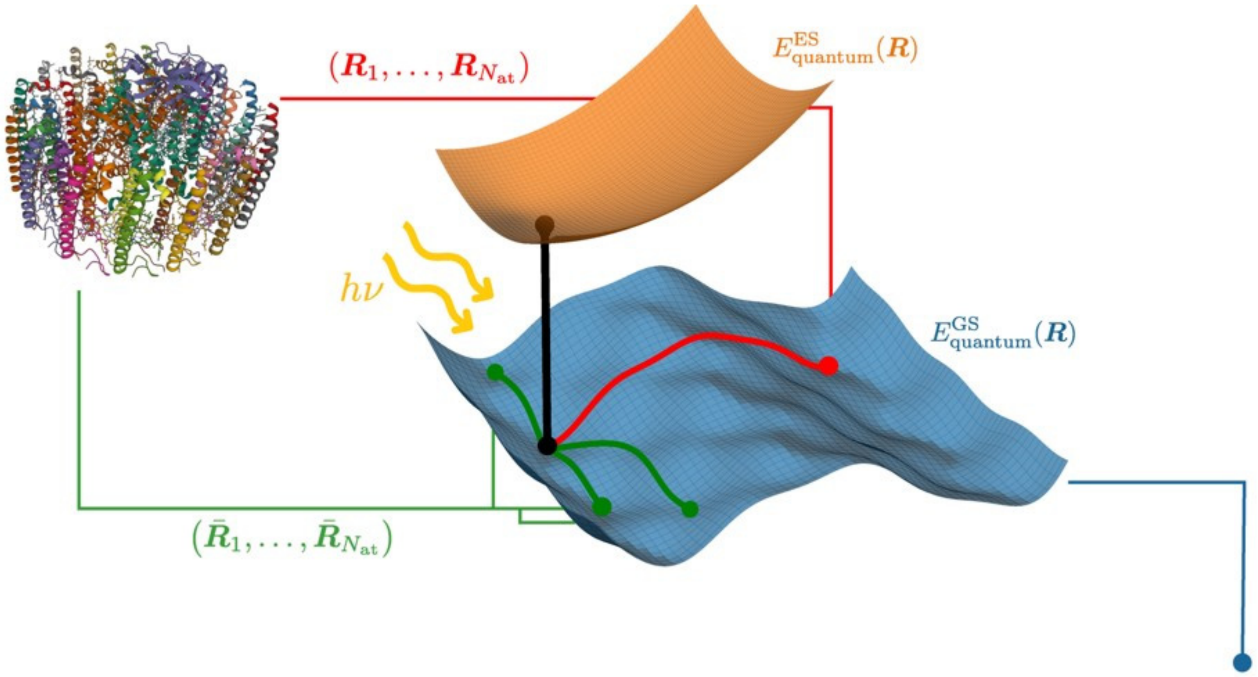

$$E^{\mathrm{GS}}_{\mathrm{classical}}(\boldsymbol{R}) = \frac{1}{2}\sum_{i=1}^{N_{\mathrm{bond}}} k_i^{(\mathrm{b})}(r_i - r_i^{\mathrm{eq}})^2 + \frac{1}{2}\sum_{j=1}^{N_{\mathrm{angle}}} k_j^{(\mathrm{a})}(\theta_j - \theta_j^{\mathrm{eq}})^2 + \sum_{m=1}^{N_{\mathrm{dihedral}}} k_m^{(d)}\left[1 + \cos\left(n\gamma_m - \gamma_m^{\mathrm{eq}}\right)\right] + \sum_{i<j}\left(\frac{C_{12}^{(ij)}}{r_{ij}^{12}} - \frac{C_6^{(ij)}}{r_{ij}^{6}}\right)$$

$E_{\mathrm{bond}}(\boldsymbol{R})$ $\quad E_{\mathrm{angle}}(\boldsymbol{R})$ $\quad E_{\mathrm{dihedral}}(\boldsymbol{R})$ $\quad E_{\mathrm{vdW}}(\boldsymbol{R})$

Therefore, from the perspective of quantum biology, "life force" or "vital energy" is not merely a physical concept but a complex informational process that permeates all levels of existence, from living cells to the human body and mind. This approach has the potential to broaden our understanding of health, treatment, and even the processes of aging and death.

In essence, life, as a complex and open system, receives quantum information from its environment and simultaneously responds to it. These responses align with the "flow of time" in such a way that life can adapt to new environmental conditions at any given moment. Thus, in quantum biology, time is not merely an external dimension but a fundamental and intrinsic element in the process of life, arising and sustaining itself in harmony and synchrony with it.

From the perspective of quantum biology, life and consciousness are understood as a set of complex processes, including quantum information transfer, vibrations, and energy frequencies. This theory allows us to explore how non-living entities can transform into living or conscious beings as information/energy in the form of specific waves and frequencies that are transmitted to them.

Source of Life and Vital Energy from the Perspective of Quantum Biology

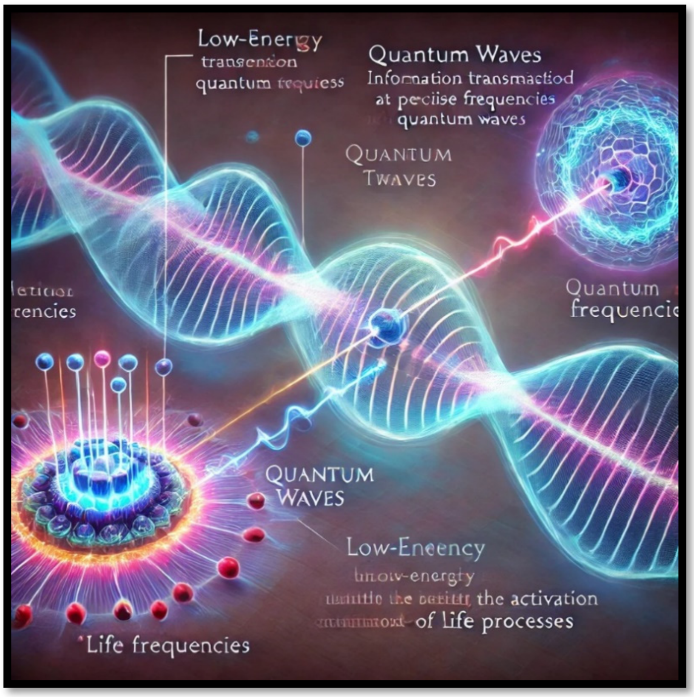

From this perspective, the primary source of life emerges from quantum energy fields that are dispersed throughout both the physical and non-physical realms of the universe. This energy is understood as "quantum information", which exhibits unique properties such as non-locality and non-linear interactions. In essence, all living beings, including cells and molecules, are interconnected with this information and possess the ability to receive and transmit it.

Graphical Representation of Key Equations for Molecular Quantum Mechanics Calculations

The electronic properties for a specific configuration of nuclei, denoted as RRR, are described by the electronic Hamiltonian operator, which is defined in the upper left corner of the diagram. The molecular Hamiltonian operator is derived by eliminating the kinetic energy of the nuclei using the fixed nuclei approximation. This approximation simplifies the complex interactions and provides a clearer representation of the electronic structure and behavior within molecular systems.

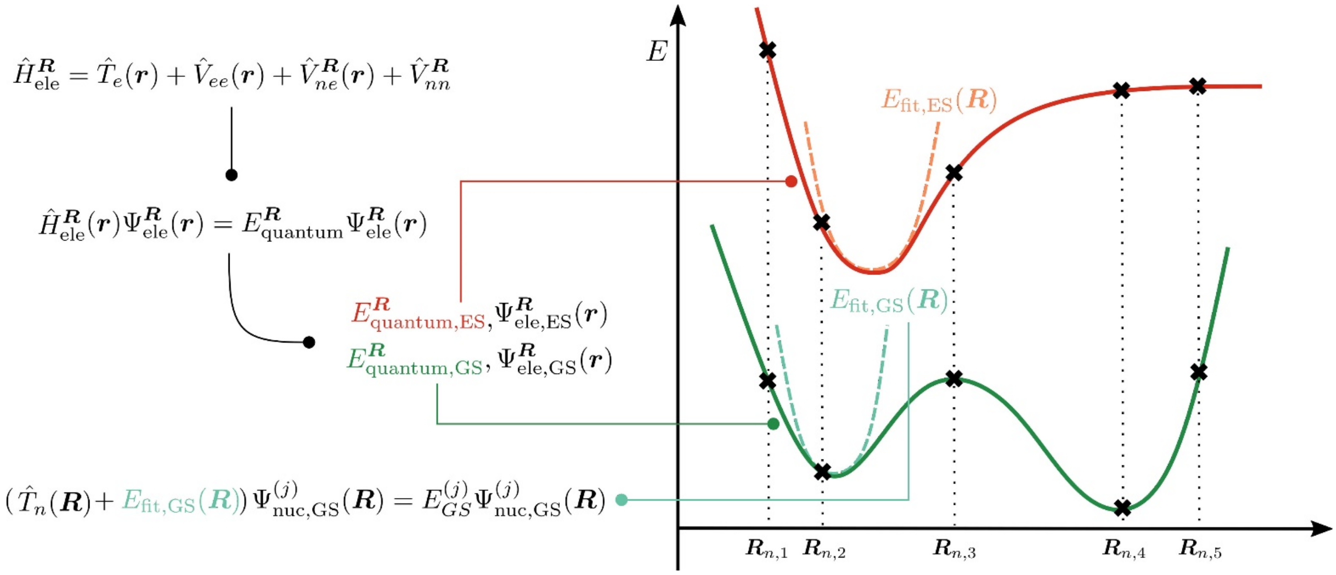

Mechanism of Information/energy Transfer

The transfer of information/energy occurs through quantum waves and vibrations. This information is transmitted to entities at specific frequencies that can activate life processes within an organism. It is hypothesized that information/energy at the quantum level is conveyed with extremely low energy and at precise, synchronized frequencies. These frequencies fall within a specific range of the electromagnetic wave spectrum, commonly referred to as “life frequencies”.

Frequencies and Wavelengths

The frequencies that transmit vital quantum information generally fall within the lower range, typically varying between 10^9 and 10^12 Hz (gigahertz to terahertz). Due to their longer wavelengths and lower energy levels, these waves enable

biological systems to interact naturally with them, facilitating the exchange of information and energy within living organisms.

Mechanism of Life Creation in Inanimate Objects

In quantum biology, life is essentially created by transferring quantum information to inanimate objects. This information can be conveyed through electromagnetic waves or even subatomic particles like photons and electrons. These particles and waves, with specific vibrations and frequencies, enter molecular and cellular structures, thereby regulating and guiding biological processes. These interactions can activate vital mechanisms in cells or even non-living materials.

From the perspective of quantum mechanics, biological systems consist of molecular interactions that are interconnected, producing complex patterns. These molecules must cross quantum energy barriers to form new structures.

In quantum calculations related to Assembly Theory, it is crucial to consider Heisenberg's uncertainty principle as, at subatomic levels, the position and energy of molecules cannot be precisely determined. This implies that the probability of molecules existing in specific assembled states is governed by quantum wave principles.

Quantum Biological Interpretation of Resonance Between Life Data and Asymmetric Quantum Vibrations in Water

In quantum biology, water is considered one of the primary carriers of quantum life information. Due to its unique structure, water molecules have the capability to store, retain, and transfer quantum information. Asymmetric quantum vibrations in water imply that quantum waves at specific frequencies can resonate with the molecular structure of water, encoding and transferring information/energy within it.

$$\frac{\sigma^2\Psi}{\sigma x^2}+\frac{\sigma^2\Psi}{\sigma y^2}+\frac{\sigma^2\Psi}{\sigma z^2}+\frac{8\pi^2 m}{h^2}(E-V)\Psi=0$$

Resonance Process

Quantum resonance occurs when the frequencies of quantum vibrations are in complete harmony with the natural frequencies of water molecules. This resonance enables water molecules to efficiently absorb and store information/energy. These quantum-transferred life data can activate vital functions such as cell repair, immune system enhancement, and nervous system strengthening.

Wavelengths and Frequencies of Information/energy Transfer

Information/energy is transferred in the form of quantum waves with specific wavelengths and frequencies. These waves generally lie within the lower range of the electromagnetic spectrum, typically between 10^9 and 10^12 Hz (gigahertz to terahertz). Due to their longer wavelengths and lower energy levels, these frequencies allow direct interaction with water molecules. The table below shows the relationship between wavelengths, key frequencies, and the quantum resonance process in water.

Unique Properties of Water Molecules:

Unique Properties of Water Molecules
The water molecule (H_2O), due to its polar structure and hydrogen bonds, can form complex networks of molecules capable of storing information. Studies have shown that the configuration of hydrogen bonds changes rapidly and vibrates at frequencies in the terahertz (THz) range. This vibrational behavior provides a suitable foundation for the transfer and storage of quantum information.

Quantum Vibrations and Resonance in Water Molecules

Molecular vibrations in water, due to hydrogen bonds, can resonate with specific quantum waves. In particular, asymmetric vibrations imply that certain specific frequencies of quantum waves can influence the molecular structure of water, thereby encoding quantum information within it. This phenomenon enables the use of water as a carrier of

information/energy. Schrödinger's famous equation can illustrate how the vibrational states of water molecules are related to quantum information.

Quantum Field Theory and the Role of Water in Information Storage
Quantum field theory clearly demonstrates that quantum fields can influence water molecules, transforming them into carriers of information. A classic example of this theory is found in the work of Del Giudice and Preparata on coherent water, where water molecules can act coherently and store and transfer quantum information.

Computational Models and Quantum Simulations
Computer simulations of water at the quantum scale, particularly using density functional theory (DFT), have shown that water molecules, due to complex electronic interactions and hydrogen bonds, can store and transfer quantum information. These models precisely predict the relationship between molecular structures and quantum states associated with information transfer.

Wavelengths and Frequencies Associated with Quantum Resonance in Water

Parameter	Frequency (Hz)	Wavelength (m)	Energy (eV)	Resonance Type
Quantum Vital Data	10^9 - 10^10	0.03 - 0.3	10^-5 - 10^-6	Resonance at Cellular Level
Molecular Vibrations of Water	10^10 - 10^11	0.003 - 0.03	10^-6 - 10^-7	Resonance at Molecular Level
Asymmetric Frequencies	10^11 - 10^12	0.0003 - 0.003	10^-7 - 10^-8	Resonance at Sub-Molecular Level

Concept of Coherence and Decoherence in Water

In quantum biology studies, water molecules can act coherently with quantum waves. Coherence means that the molecules operate in an orderly quantum state. In contrast, decoherence refers to the loss of this coherence. Under certain conditions, water can maintain longer coherent states, allowing it to store quantum information effectively.

Mathematical Proof of Water Memory Capacity and Reasoning

The memory of water relates to its capacity to retain quantum information, which can be described through equations related to coherence and hydrogen bonds.

- **III** represents the information capacity.
- **NNN** represents the number of coherent hydrogen bonds in a given volume of water.
- **SSS** represents the entropy of the system, indicating the amount of information stored.

The coherence of hydrogen bonds plays a crucial role in water's ability to store information, with higher coherence suggesting better retention of quantum information.

The Concept of Coherence and Decoherence in Water

The concept of coherence and decoherence in water is a significant topic in quantum biology and can help explain how quantum information is stored and transmitted. This section provides a scientifically detailed explanation of these phenomena.

In quantum biology, water molecules can act coherently with quantum waves. Coherence refers to a state where molecules operate in a synchronized quantum state, allowing the effective transfer and storage of quantum information. In contrast, decoherence is the loss of this synchrony, often caused by environmental factors like temperature and fluctuations.

Coherence Conditions and Its Effect on Quantum Information
Water can maintain coherent states for extended periods under specific conditions, enabling it to store and transfer quantum information.

Proof Formulas:
1. Schrödinger's Equation:
$\widehat{\Psi} H \Psi = E \Psi$
This equation describes the energy state and information of water molecules in interaction with quantum waves.

2. Wave Functions of Water Molecules:
These wave functions originate from hydrogen bonds and have the potential to store and transmit information as resonant frequencies.

3. Quantum Model of Hydrogen Bonds:
$\nu = 1/(2\pi) * \sqrt{(k/m)}$
This equation defines the vibrational behavior of water molecules and hydrogen bonds.

Conclusion
In a coherent state, quantum information can be transferred and stored between water molecules due to their synchronization, creating a network of stable hydrogen bonds. When this state is disrupted (e.g., due to temperature increase or water evaporation), hydrogen bonds break, and the water's memory is lost.

This concept aids a deeper understanding of how quantum information functions in water and its effects on quantum biology.

Quantum Information Storage Capacity of Water

The capacity for storing quantum information in water is related to its ability to retain quantum information, which can be described through equations related to coherence and hydrogen bonds.

1. Capacity for Storing Quantum Information
The capacity for storing quantum information in water is a function of the number of hydrogen bonds and the coherent states present. This capacity can be approximated by the following relation:

$I = N \times S$

- I: Information capacity.
- N: Number of coherent hydrogen bonds in a specific volume of water.
- S: The entropy of the system, representing the amount of stored information.

2. Breaking Hydrogen Bonds During the Evaporation Process
During evaporation, the kinetic energy of the molecules increases, leading to the breaking of hydrogen bonds. This process causes N to approach zero, represented as follows:

$\lim_{T \to \infty} N = 0$

where T represents temperature, and as temperature increases during the evaporation process, the number of hydrogen bonds decreases to zero. This indicates the complete erasure of the water's memory.

3. The Condensation and Reconstruction of Bonds
After condensation, water molecules reform hydrogen bonds; however, these new bonds do not possess the previous coherent state. This phenomenon can be likened to returning to a more random state after condensation:
$N_{new} \neq N_{original}$

where N_{new} is the number of new bonds that do not contain prior information.

To evaluate the effect of physical changes in energy stored in the water, 3 samples of water with the code of “Dei1_66h” (deionized water of the pyramid floor after 3 days) with energy of 17.9 ± 0.8 mJ were exposed to different physical conditions such as cold (9 hours freezing), heat (5 minutes boiling), and other electromagnetic radiation such as 10 hours of exposure to a Wi-Fi and 1 minute to a microwave device. Afterwards, the energy of the samples again were measured. The results are shown in Table 7. In order to protect the water samples against external waves, during transformation they were kept in the aluminum coating around the flasks and inside an anti-radiation aluminum bag.

Table 7: Effect of physical changes in energy stored in water Dei1_66h

physical changes	Mean ±SD mJ
10 h exposing to WiFi	17.8 ± 2.0
after 5 min boiling	17.5 ± 1.9
after 9 h freezing	18.3 ± 1.8
1 min in microwave	16.1 ± 1.0

Created Technology (Quantum Carbon Embryo Named Fibonacci Atlantis)

The design and construction of the Fibonacci Atlantis quantum carbon unit were completed after 16,200 stages of technical failures and 122 million simulated scenarios using a genetic algorithm in COMSOL Multiphysics software.

Genetic Algorithm: The genetic algorithm is the most well-known optimization method used to determine the required layers in a composite structure with specific properties. The general flow of this algorithm is illustrated in the figure below.

This sophisticated approach enabled the creation of a quantum carbon unit with optimized characteristics, designed for enhanced coherence and stability, ensuring its functionality in quantum information transfer and related applications.

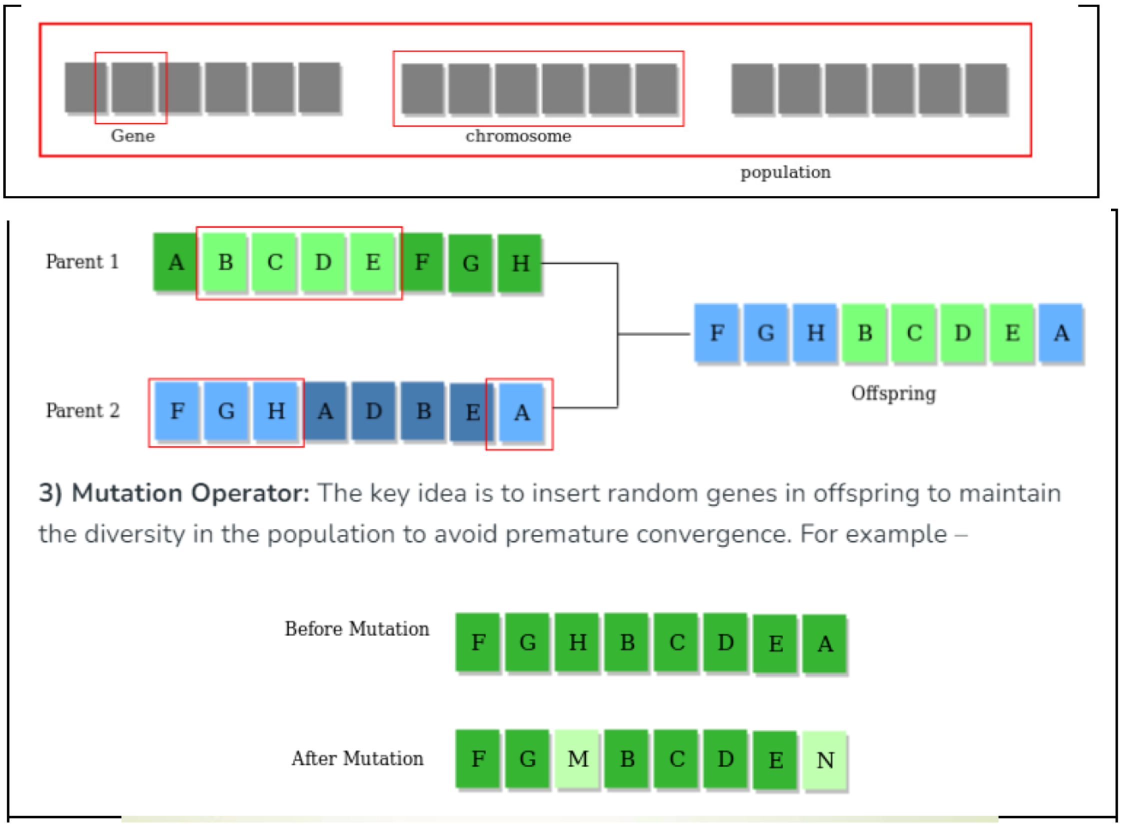

- **Step1**: Represent the problem variable domain as a chromosome of a fixed length, choose the size of a chromosomes population *N*, the crossover probability *P*, and the mutation probability *Pm*.
- **Step2** Define a fitness function to measure the performance, or fitness, of a individual chromosome in the problem domain. The fitness function establishes the basis for selecting chromosomes that will be mated during reproduction.
- **Step3** Randomly generate an initial population of chromosomes of size N: x_1, x_2, ……x_N.

Created Technology (Quantum Carbon Embryo Named Fibonacci Atlantis)

The energy and quantum information source in the designed unit comes from natural phenomena such as cosmic rains, Earth's natural electromagnetic energies, solar radiation, and other unknown sources. This unit effectively transfers these natural energies to water using the principles of coherence and quantum superposition.

3.2. Sample Preparation: Water samples were prepared after being exposed to the quantum unit for 72 hours and were used to study its effects on neuronal and immune cells.

In the simulated and hardware-produced unit, quantum biological life information was received from an unknown source of life in nature at a wavelength of 300 - 3000 nm with energy of 0.414-4.14 eV. This information was amplified 6400 times and transferred to the quantum tensile vibration frequency of water, which had a wavelength of 120 nm, and energy of 14.4 eV. The information/energy was then added to the hexagonal water molecules.

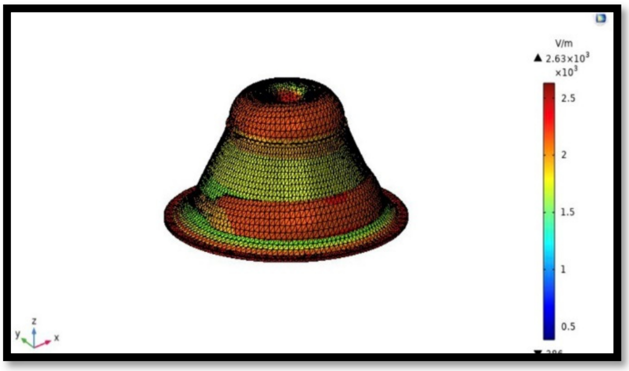

1.Fibonacci Atlantis Simulation in COMSOL Multiphysics Software

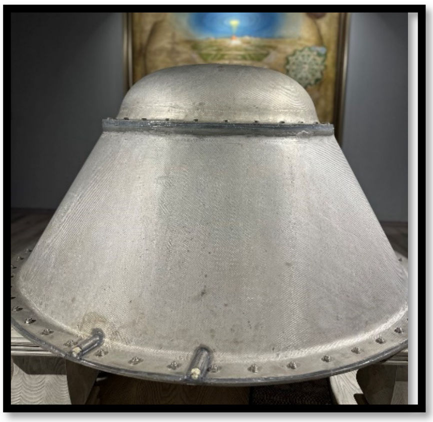

2.Carbon Quantum Embryo Produced by Fibonacci Atlantis

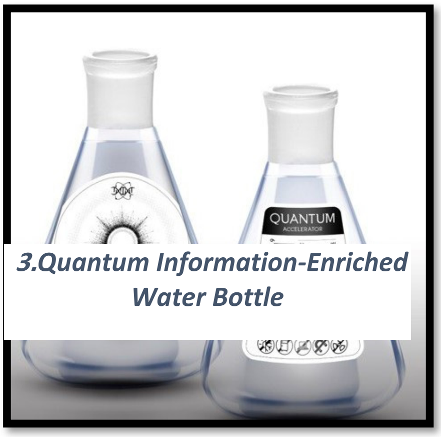

3.Quantum Information-Enriched Water Bottle

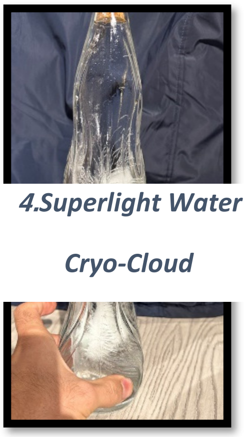

4.Superlight Water

Cryo-Cloud

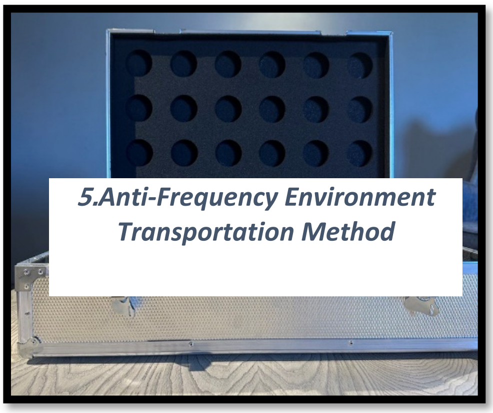

5.Anti-Frequency Environment Transportation Method

Method and Equipment for Water Measurement

In this study, energy measurements were conducted using the Electrophotonic Imaging (EPI) electromagnetic imaging technique. This technique has a historical background in Eastern countries and is based on the Kirlian effect. The Kirlian effect, originally used in early studies analyzing Kirlian spectra, has been applied as a unique energy emission effect with maximum brightness for medical applications and disease diagnosis.

In this technique, electromagnetic fields are observed by separating the electric and magnetic spectrum for Gas Discharge Visualization (GDV) using sensitive CCD detectors. This is used as a method to collect radiative electro-photonic signals from living organisms by applying high-frequency electrical stimulation.

In the EPI imaging technique, after electrical stimulation of the environment through the transmission of extremely high-frequency electromagnetic waves (primarily in the range of 40-70 GHz) at low intensities (usually 10 mW/cm² or less), the dielectric constant of the environment decreases, leading to reduced environmental resistance and the occurrence of electrical discharge, resulting in the observation of the GDV phenomenon.

By collecting the electro-photons produced by the Kirlian effect and analyzing the digital images, the electric field on the surface or in the surrounding environment could be calculated using the glow image (GI) spectral emission. This allowed for the determination of the stored energy, which was used to assess the physiological status of a person or living organism.

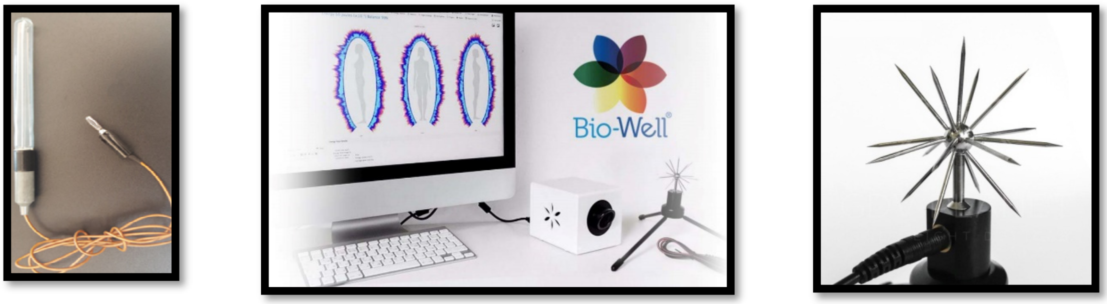

6.Measurement Methods and Equipment

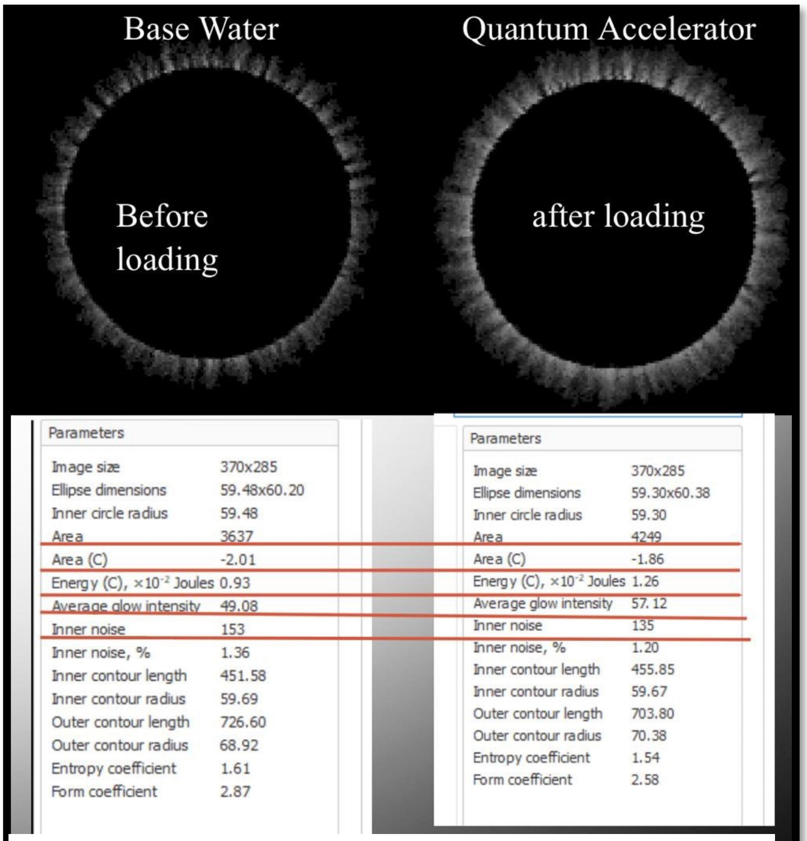

7.Holographic Energy and Quantum Information of a Water Droplet Before and After Exposure to the Fibonacci Atlantis

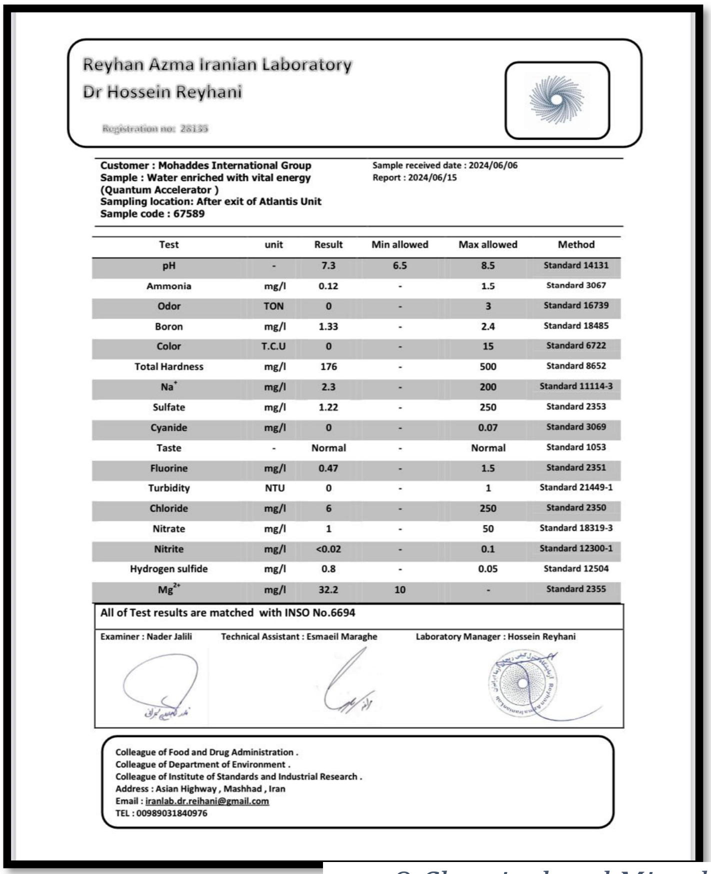

Reyhan Azma Iranian Laboratory
Dr Hossein Reyhani
Registration no: 28135

Customer : Mohaddes International Group
Sample : Water enriched with vital energy (Quantum Accelerator)
Sampling location: After exit of Atlantis Unit
Sample code : 67589
Sample received date : 2024/06/06
Report : 2024/06/15

Test	unit	Result	Min allowed	Max allowed	Method
pH	-	7.3	6.5	8.5	Standard 14131
Ammonia	mg/l	0.12	-	1.5	Standard 3067
Odor	TON	0	-	3	Standard 16739
Boron	mg/l	1.33	-	2.4	Standard 18485
Color	T.C.U	0	-	15	Standard 6722
Total Hardness	mg/l	176	-	500	Standard 8652
Na^+	mg/l	2.3	-	200	Standard 11114-3
Sulfate	mg/l	1.22	-	250	Standard 2353
Cyanide	mg/l	0	-	0.07	Standard 3069
Taste	-	Normal	-	Normal	Standard 1053
Fluorine	mg/l	0.47	-	1.5	Standard 2351
Turbidity	NTU	0	-	1	Standard 21449-1
Chloride	mg/l	6	-	250	Standard 2350
Nitrate	mg/l	1	-	50	Standard 18319-3
Nitrite	mg/l	<0.02	-	0.1	Standard 12300-1
Hydrogen sulfide	mg/l	0.8	-	0.05	Standard 12504
Mg^{2+}	mg/l	32.2	10	-	Standard 2355

All of Test results are matched with INSO No.6694

Examiner : Nader Jalili
Technical Assistant : Esmaeil Maraghe
Laboratory Manager : Hossein Reyhani

Colleague of Food and Drug Administration .
Colleague of Department of Environment .
Colleague of Institute of Standards and Industrial Research .
Address : Asian Highway , Mashhad , Iran
Email : iranlab.dr.reihani@gmail.com
TEL : 00989031840976

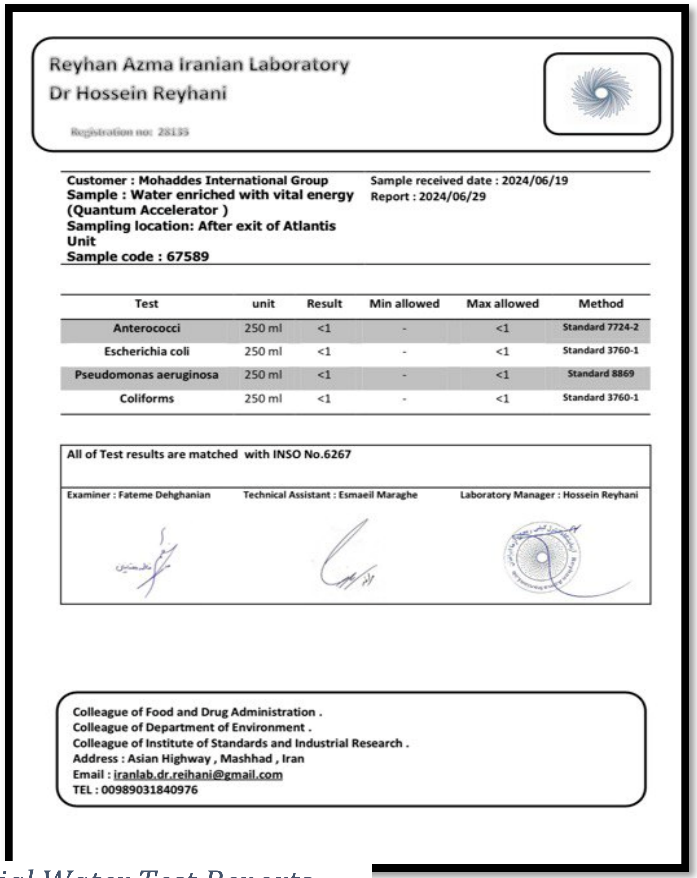

Reyhan Azma Iranian Laboratory
Dr Hossein Reyhani
Registration no: 28135

Customer : Mohaddes International Group
Sample : Water enriched with vital energy (Quantum Accelerator)
Sampling location: After exit of Atlantis Unit
Sample code : 67589
Sample received date : 2024/06/19
Report : 2024/06/29

Test	unit	Result	Min allowed	Max allowed	Method
Anterococci	250 ml	<1	-	<1	Standard 7724-2
Escherichia coli	250 ml	<1	-	<1	Standard 3760-1
Pseudomonas aeruginosa	250 ml	<1	-	<1	Standard 8869
Coliforms	250 ml	<1	-	<1	Standard 3760-1

All of Test results are matched with INSO No.6267

Examiner : Fateme Dehghanian
Technical Assistant : Esmaeil Maraghe
Laboratory Manager : Hossein Reyhani

Colleague of Food and Drug Administration .
Colleague of Department of Environment .
Colleague of Institute of Standards and Industrial Research .
Address : Asian Highway , Mashhad , Iran
Email : iranlab.dr.reihani@gmail.com
TEL : 00989031840976

8.Chemical and Microbial Water Test Reports

References on Water Memory and Measurement Methods: This section provides references on the concept of water memory and various methods used to measure it

Montagnier, L., Aissa, J., Del Giudice, E., & Lavallee, C. (2011). "DNA waves and water."

Description: This research investigates the transfer of bio-quantum information from DNA to water and demonstrates that water can store biological information through electromagnetic frequencies.

https://www.ncbi.nlm.nih.gov/pubmed/21730461

Del Giudice, E., & Preparata, G. (1998). "Coherent Dynamics in Water as a Natural Medium for Information Storage." Modern Physics Letters B.

This Article: This article is from the pioneers of the quantum memory of water theory and explores how quantum information can be stored and transferred in water.

https://www.worldscientific.com/doi/abs/10.1142/S0217984998002425

Chaplin, M. F. (2010). "Water: Its Importance to Life." Biophysics of Water.

This Research: This research examines the unique properties of water and its impact on biological systems, particularly in the context of quantum memory.

http://www1.lsbu.ac.uk/water/water.html

Bio-Well Company Bio-Well Water Sensor Manual, Ver. 10-2017 ,

Method for Measuring Information/energy in Water (Quantum Accelerator): This group uses a method for measuring Information/energy in water involving the quantum accelerator to evaluate the quantum properties and biological impact of the water

https://www.bio-well.com

Janifal Alipal, 2Razak Mohd Ali Lee, 3Ali Farzamnia; Preliminary Study of Kirlian Image in Digital Electrophotonic Imaging and its Applications; Department of Electrical and Electronics Engineering, Faculty of Engineering, University Sabah Malaysia (UMS),

https://www.researchgate.net/publication/322058094

Zeidler, M. D., et al. (2011). "Quantum mechanical studies of water molecules." Journal of Chemical Physics.

https://aip.scitation.org/doi/10.1063/1.3555382

Popov, A. M., et al. (2019). "Quantum effects in water molecular dynamics." Journal of Molecular Liquids.

Investigation of Quantum Effects on Molecular Dynamics of Water and How Quantum Information is Stored and Transferred.

https://www.sciencedirect.com/science/article/abs/pii/S0167732218311281

Jang, S. H., et al. (2015). "Quantum coherence in the water hydrogen bond network." Physical Review E.

This Article: This article examines the role of coherence in the hydrogen bond network of water and how quantum information is preserved.

https://journals.aps.org/pre/abstract/10.1103/PhysRevE.92.052119

Grigoryev, I. M. (2008). "The quantum structure of liquid water." Physics-Uspekhi.

https://ufn.ru/en/articles/2008/4/h/

Fröhlich, H. (1968). "Long-range coherence and energy storage in biological systems." Nature.

https://www.nature.com/articles/2201339a0

Berkovitch-Yellin, Z., et al. (2016). "Quantum water dynamics." Nature Communications.

https://www.nature.com/articles/ncomms12069

Saitta, A. M., et al. (2014). "Quantum tunneling in water clusters." Journal of Chemical Physics.

https://aip.scitation.org/doi/10.1063/1.4901776

References: "Quantum Biology" and Quantum Information in Biological Processes:

Schumann, W. O., & König, H. (1954). "Über die Beobachtung von atmosphärischen Eigenschwingungen."

The First Article on Schumann Resonance: The first article on Schumann resonance shows how Earth's natural frequencies in the range of 7.83 Hz can resonate with biological systems.

https://www.sciencedirect.com/science/article/pii/S0031917315303097

Quantum Computing for Molecular Biology

Quantum Computing for Molecular Biology: This explores the applications of quantum computing technology in various areas of molecular biology.

https://chemistry-europe.onlinelibrary.wiley.com/doi/10.1002/cbic.202300120

Zeh, H. D. (2003). "The Meaning of Decoherence." In: *Quantum Theory: Concepts and Methods*. Springer

Quantum Computing for Molecular Biology: This explores the applications of quantum computing technology in various areas of molecular biology.

https://link.springer.com/book/10.1007/978-3-642-18924-9

Tegmark, M. (2000). "Importance of Quantum Coherence in Biological Processes." ***Physical Review E*****, 61(4), 4194-4206.**

This Article: This article examines the role of quantum entanglement in biological processes and its potential in maintaining homeostasis

https://journals.aps.org/pre/abstract/10.1103/PhysRevE.61.4194

Fleming, G. R., & Durrant, J. R. (2009). "Quantum Mechanics and the Nature of Life." ***Nature Reviews Chemistry*****, 1(1), 7-18.**

This Article: This article explores how quantum mechanics affects biological systems and the concept of life

https://www.nature.com/articles/nchem.2009.5

Hagan, S., et al. (2009). "Quantum Coherence in Photosynthetic Systems." ***Nature Chemistry*****, 1(9), 781-790.**

This Article: This article examines the use of quantum entanglement in photosynthetic systems and its role in energy transfer.

https://www.nature.com/articles/nchem.2009.31

Kauffman, S. A. (2000). "Investigations." Oxford University Press.

This Book: This book explores theories related to the emergence and evolution of life and the role of information in biological processes

https://global.oup.com/academic/product/investigations-9780195131604

Bialynicki-Birula, I. (1996). "Quantum Information and Its Implications." ***Annals of Physics*****, 248(1), 121-146.**

This Article: This article explores the concepts of quantum information and its impact on physics and biology.

https://www.sciencedirect.com/science/article/pii/S0003491696900566

Wang, H., & Hiller, R. (2011). "Quantum Information Processing in Living Systems." ***Physics Reports*****, 499(1), 1-54.**

A Comprehensive Article: A comprehensive article on quantum information processing in living systems and its effects on health and life

https://www.sciencedirect.com/science/article/pii/S0370157311001985

Miller, J. (2003). "Quantum Mechanics in Biological Systems." ***Journal of Theoretical Biology*****, 224(1), 95-103.**

This Article: This article explores quantum mechanics and its impact on biological and chemical processes

https://www.sciencedirect.com/science/article/pii/S0022519303000194

Ghirardi, G. C., et al. (1986). "Unified Dynamics for Microscopic and Macroscopic Systems." ***Physical Review D*****, 34(2), 470-491.**

This Article: This article examines unified dynamics for microscopic and macroscopic systems and their impacts on biology.

https://journals.aps.org/prd/abstract/10.1103/PhysRevD.34.470

Schrodinger, E. (1944). "What is Life? The Physical Aspect of the Living Cell." Cambridge University Press.

This Book: This book explores the physical aspects of life and how they relate to physical and quantum laws.

https://www.cambridge.org/core/books/abs/what-is-life/5E4D77D9D69BB3B5A4C60BCE2A1036C0

Thesis officially completed.
Prepared by **International Group of Ali Akbar Mohades**
Website: www.intgraam.com

With utmost respect and appreciation,
Ali Akbar Mohades
