# Supplementary material for: Quantum Health Accelerator® Ameliorates CFA-Induced Animal Model of Rheumatoid Arthritis: Investigating the Role of Immunomodulatory and Anti-Oxidative Effects
Source: Brain Sci. 2025 Feb 23;15(3):232. doi: 10.3390/brainsci15030232 (PMC11940038; doi:10.3390/brainsci15030232)
Supplement: Supplementary file 1 [file brainsci-15-00232-s001.zip › Supp file for conversion.pdf]

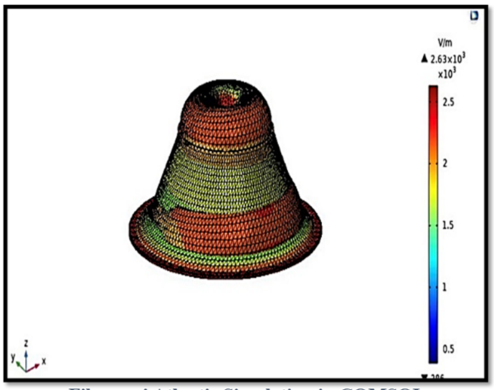

Fibonacci Atlantis Simulation in COMSOL Multiphysics Software

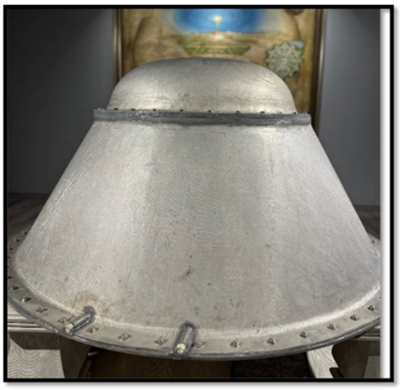

Carbon Quantum Embryo Produced by Fibonacci Atlantis

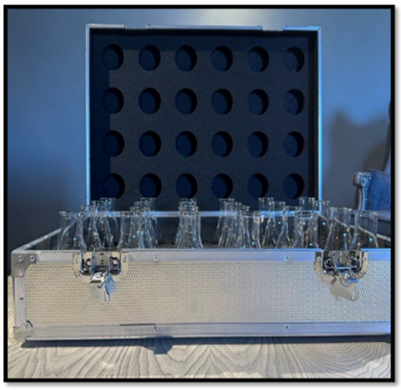

Anti-Frequency Environment Transportation Method

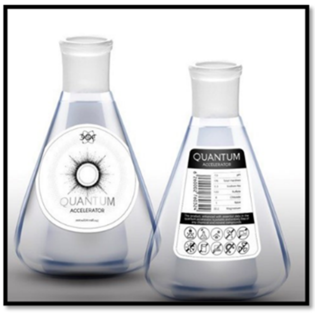

Quantum Information-Enriched Water Bottle

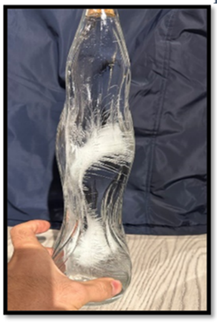

Superlight Water Cryo-Cloud

A)

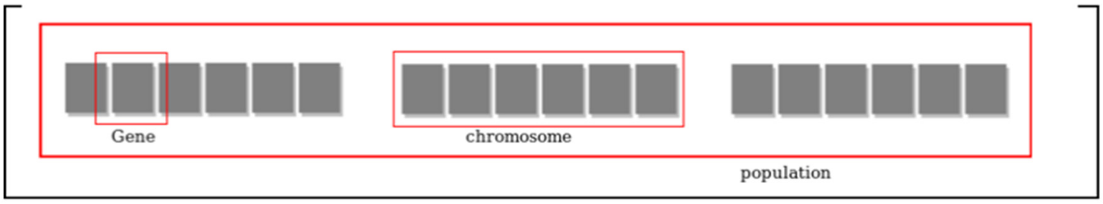

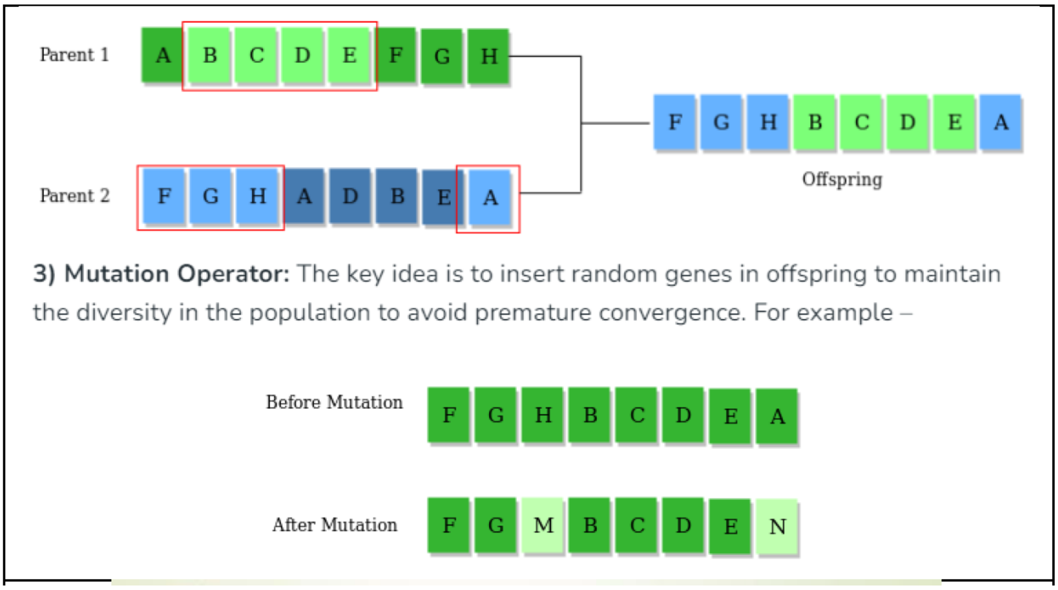

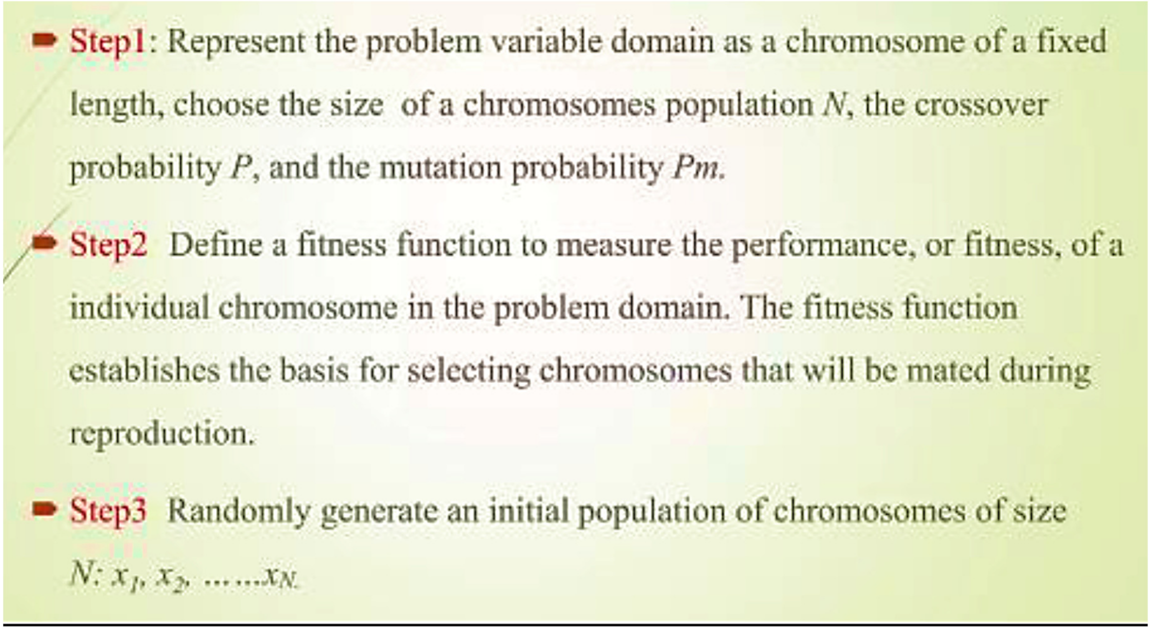

Figure S1. Fibonacci Atlantis Simulation in COMSOL Multiphysics Software and Carbon Quantum Embryo Produced by Fibonacci Atlantis. A genetic algorithm is used to create a quantum carbon unit with optimized characteristics that are designed for enhanced coherence and stability, ensuring its functionality in quantum information transfer and related applications. The genetic algorithm is the most well-known optimization method used to determine the required layers in a composite structure with specific properties.

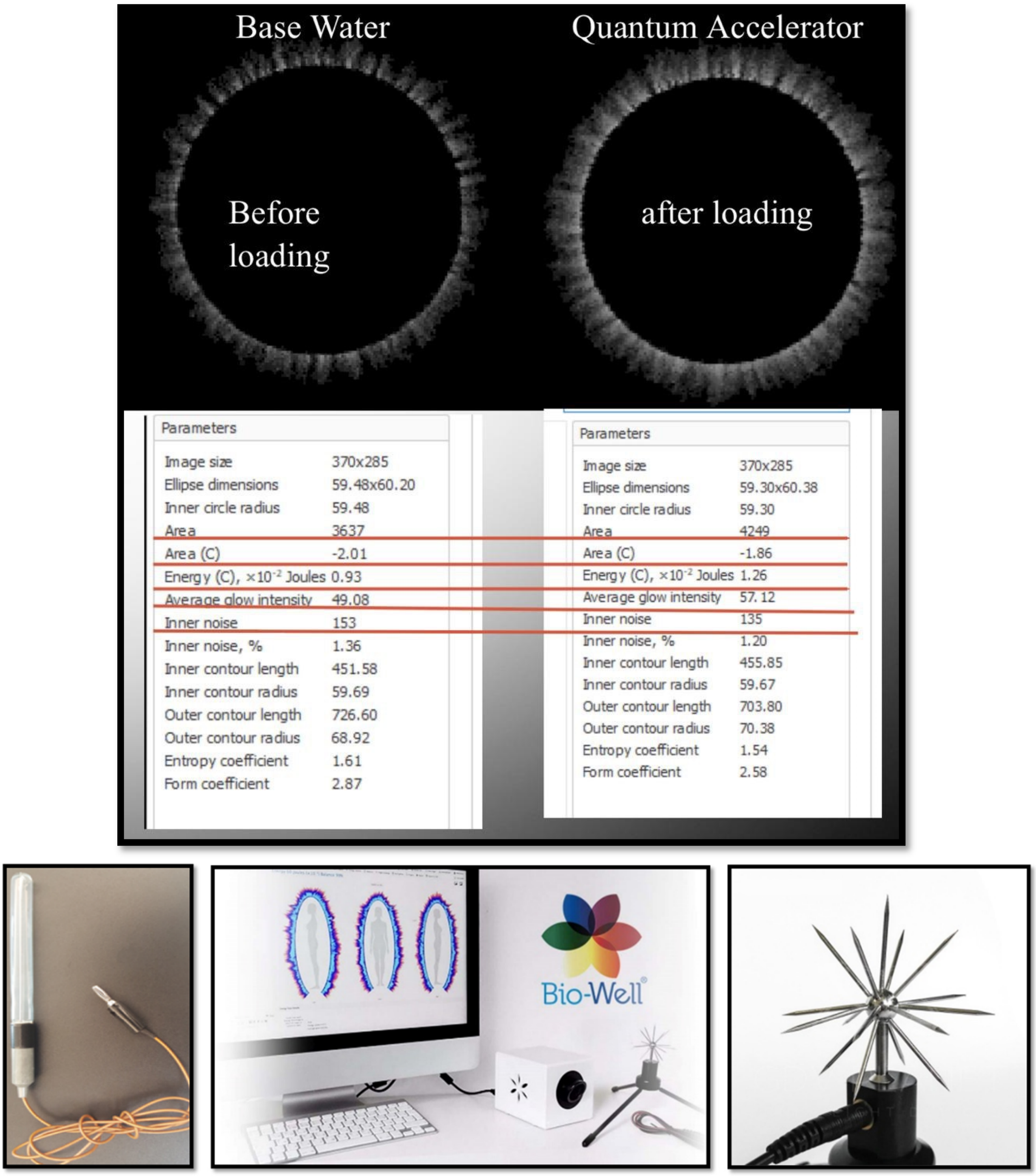

Figure S2. Holographic Energy and Quantum Information of a Water Droplet Before and After Exposure to the Fibonacci Atlantis along with the measurment tools.

Radiation spectrum by collecting the electron photons produced by the Kirlian phenomenon and analyzing their digital images. By collecting the electron photons produced by the Kirlian phenomenon and analyzing their digital images, the electric field on the surface of an object or in the surrounding environment can be calculated using the radiation spectrum in GI (Glow Image) images, and the energy stored in these images can be used to determine the physiological state of an individual or living organism.

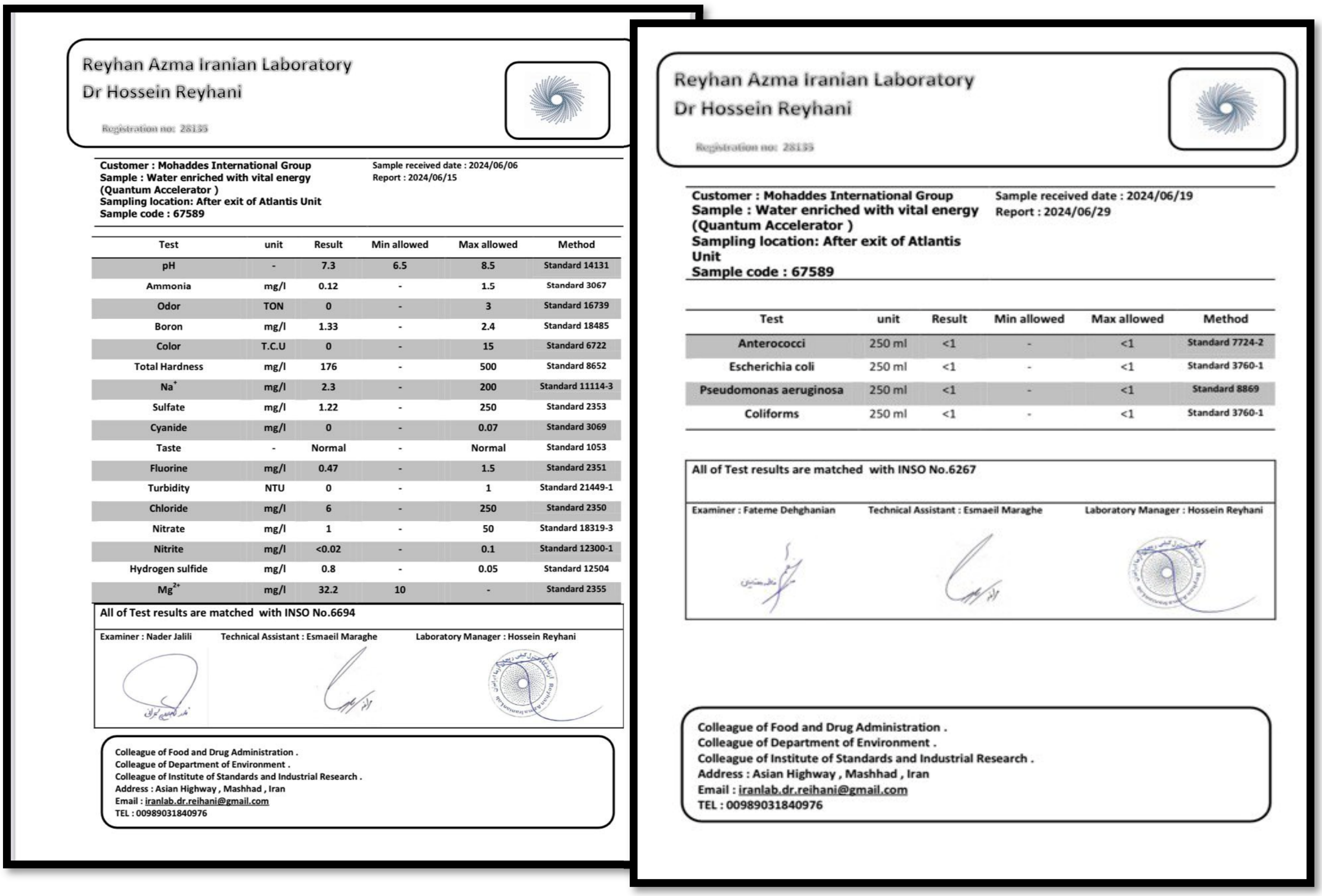

Reyhan Azma Iranian Laboratory
Dr Hossein Reyhani
Registration no: 28135

Customer : Mohaddes International Group
Sample : Water enriched with vital energy (Quantum Accelerator)
Sampling location: After exit of Atlantis Unit
Sample code : 67589
Sample received date : 2024/06/06
Report : 2024/06/15

Test	unit	Result	Min allowed	Max allowed	Method
pH	-	7.3	6.5	8.5	Standard 14131
Ammonia	mg/l	0.12	-	1.5	Standard 3067
Odor	TON	0	-	3	Standard 16739
Boron	mg/l	1.33	-	2.4	Standard 18485
Color	T.C.U	0	-	15	Standard 6722
Total Hardness	mg/l	176	-	500	Standard 8652
Na^+	mg/l	2.3	-	200	Standard 11114-3
Sulfate	mg/l	1.22	-	250	Standard 2353
Cyanide	mg/l	0	-	0.07	Standard 3069
Taste	-	Normal	-	Normal	Standard 1053
Fluorine	mg/l	0.47	-	1.5	Standard 2351
Turbidity	NTU	0	-	1	Standard 21449-1
Chloride	mg/l	6	-	250	Standard 2350
Nitrate	mg/l	1	-	50	Standard 18319-3
Nitrite	mg/l	<0.02	-	0.1	Standard 12300-1
Hydrogen sulfide	mg/l	0.8	-	0.05	Standard 12504
Mg^{2+}	mg/l	32.2	10	-	Standard 2355

All of Test results are matched with INSO No.6694

Examiner : Nader Jalili
Technical Assistant : Esmaeil Maraghe
Laboratory Manager : Hossein Reyhani

Colleague of Food and Drug Administration .
Colleague of Department of Environment .
Colleague of Institute of Standards and Industrial Research .
Address : Asian Highway , Mashhad , Iran
Email : iranlab.dr.reihani@gmail.com
TEL : 00989031840976

Reyhan Azma Iranian Laboratory
Dr Hossein Reyhani
Registration no: 28135

Customer : Mohaddes International Group
Sample : Water enriched with vital energy (Quantum Accelerator)
Sampling location: After exit of Atlantis Unit
Sample code : 67589
Sample received date : 2024/06/19
Report : 2024/06/29

Test	unit	Result	Min allowed	Max allowed	Method
Anterococci	250 ml	<1	-	<1	Standard 7724-2
Escherichia coli	250 ml	<1	-	<1	Standard 3760-1
Pseudomonas aeruginosa	250 ml	<1	-	<1	Standard 8869
Coliforms	250 ml	<1	-	<1	Standard 3760-1

All of Test results are matched with INSO No.6267

Examiner : Fateme Dehghanian
Technical Assistant : Esmaeil Maraghe
Laboratory Manager : Hossein Reyhani

Colleague of Food and Drug Administration .
Colleague of Department of Environment .
Colleague of Institute of Standards and Industrial Research .
Address : Asian Highway , Mashhad , Iran
Email : iranlab.dr.reihani@gmail.com
TEL : 00989031840976

Figure S3. Chemical and Microbial Water Test Reports

Mashhad University of Medical Sciences

School of Medicine- Mashhad University of Medical Sciences

Research Ethics Committees Certificate

Approval ID:	IR.MUMS.MEDICAL.REC.1400.297	Approval Date:	2021-07-06
Evaluated by:	Research Ethics Committees of School of Medicine- Mashhad University of Medical Sciences		
Status:	Approved		
Approval Statement:	The project was found to be in accordance to the ethical principles and the national norms and standards for conducting Medical Research in Iran.<br>Notice:<br>1. Although the proposal has been approved by the Biomedical Research Ethics Committee, meeting the professional and legal requirements is the sole responsibility of the PI and other project collaborators.<br>2. This certificate is reliant on the proposal/documents received by this committee on 2021-07-06. The committee must be notified by the PI as soon as the proposal/documents are modified.		
Proposal Title:	Evaluation of the anti-inflammatory and anti-oxidant effects of Energetic water in complete Freund's adjuvant-induced rats' model of arthritis		
Principal Investigator:	Name: Vahid Reza Askari<br>Email: AskariV@mums.ac.ir		

Dr. Mahmood Tara
Committee Director
School of Medicine- Mashhad University of Medical Sciences

Dr. Maliheh Dadgar Moghadam
Committee Secretary
School of Medicine- Mashhad University of Medical Sciences

Figure S4. All experiments and procedures were organized according to the *NIH's Guideline for the Care and Use of Laboratory Animals*, accompanying the approval from the *Animal Ethics Committee of Mashhad University of Medical Sciences* (approval no. 4000351, IR.MUMS.MEDICAL.REC.1400.297, Date: 2021-7-6).
